# CDK4/6 inhibitors versus PI3K/AKT/mTOR inhibitors in women with hormone receptor-positive, HER2-negative metastatic breast cancer: An updated systematic review and network meta-analysis of 28 randomized controlled trials

**DOI:** 10.3389/fonc.2022.956464

**Published:** 2022-08-24

**Authors:** Hangcheng Xu, Yan Wang, Yiqun Han, Yun Wu, Jiayu Wang, Binghe Xu

**Affiliations:** Department of Medical Oncology, National Cancer Center/National Clinical Research Center for Cancer/Cancer Hospital, Chinese Academy of Medical Sciences and Peking Union Medical College, Beijing, China

**Keywords:** CDK4/6 inhibitors, PI3K/AKT/mTOR inhibitors, hormone receptor-positive, HER2-negative, metastatic breast cancer, network meta-analysis

## Abstract

**Background:**

Updated evidence was required to compare the efficacy and safety of cyclin-dependent kinases 4 and 6 (CDK4/6) inhibitors and phosphatidylinositol 3-kinase (PI3K)/protein kinase B (AKT)/mammalian target of rapamycin (mTOR) inhibitors for patients with hormone receptor-positive and HER2-negative metastatic breast cancer.

**Methods:**

A systematic review and network meta-analysis was conducted utilizing data from randomized controlled trials (RCTs) that contained interventions of CDK4/6 inhibitors or PI3K/AKT/mTOR inhibitors. Progression-free survival (PFS), overall survival (OS), and treatment-related adverse events (TRAEs) were primary outcomes of interest. Pooled hazard ratios (HRs) and odds ratios (ORs) with 95% credible intervals (CrIs) were used to assess the survival outcomes and safety profiles, respectively.

**Results:**

A total of 28 RCTs with 12,129 participants were included. Pooled analysis showed that CDK4/6 inhibitors significantly prolonged PFS than PI3K/AKT/mTOR inhibitors (HR, 0.81; 95% CrI, 0.69–0.94), whereas no significant differences were detected regarding OS. After balancing the treatment lines and metastatic sites, the superiority of CDK4/6 inhibitors only appeared in the visceral and non-visceral subgroups. Among CDK4/6 inhibitors, abemaciclib was significantly better than others in ≥3 grade neutropenia (OR, 0.04; 95% CrI, 0.01–0.15). The incidence of stomatitis and digestive disorders was different among diverse kinds of PI3K/AKT/mTOR inhibitors. Discrepancies appeared regarding TRAEs of hepatotoxicity, diarrhea, and hyperglycemia among different interventions.

**Conclusions:**

CDK4/6 inhibitors showed better efficacy in PFS, but the benefits disappeared when taking treatment line into consideration. Specific and discrepant safety profiles were found in two categories of agents.

**Systematic Review Registration:**

https://www.crd.york.ac.uk/PROSPERO, identifier CRD42022321172.

## Introduction

Surpassing lung cancer, breast cancer has become the most common malignancy diagnosed worldwide, with 2.3 million new cases in 2020 (1). According to the status of hormone receptor, including estrogen receptor (ER) and progesterone receptor (PR), and human epidermal growth factor 2 (HER2), breast cancer is categorized into distinctive molecular subtypes, guiding the diagnosis and treatment for decades (2). For hormone receptor-positive/HER2-negative subtype, which accounts for a large proportion of breast cancer, endocrine therapy (ET), including aromatase inhibitors (AIs), selective ER modulators (SERMs), and selective ER down-regulators (SERDs), is the bedrock (3, 4). However, the resistance to ET, either primary or secondary, poses a barrier for the subsequent treatment options (5). The emergence of cyclin-dependent kinases 4 and 6 (CDK4/6) inhibitors and phosphatidylinositol 3-kinase (PI3K)/protein kinase B (AKT)/mammalian target of rapamycin (mTOR) inhibitors has addressed this issue to some extent. 

CDK4/6, as a subgroup of serine/threonine kinases, promote cell cycle regulation by phosphorylating retinoblastoma (Rb) protein after interacting with cyclin D and then initiate the cell cycle transition from G1 phase to S phase (6). CDK4/6 overexpression is frequently encountered in hormone receptor-positive breast cancer and the highly selective inhibitors of which combined with ET are standard care of first-line treatment for these patients in advanced-stage (7). Other than CDK4/6 signaling pathway, PI3K/AKT/mTOR (or PAM) signaling pathway was also proved to be a major one in disease recurrence or progression (8). Studies indicated that the PAM pathway was essential for cellular proliferation and metabolism, and the activation of which was found in up to 70% of the breast cancer (9). There also existed elaborate cross-talk between the PAM and estrogen-mediated signaling pathway (10). It is estimated that approximately 40% to 50% of patients with hormone receptor-positive/HER2− breast cancer have aberrant activation of *PIK3CA* (encoding the p110α isoform of PI3K) (11, 12), which is closely correlated with ET resistance. Currently, pan or selective PI3K/AKT/mTOR inhibitors are promising agents for patients with hormone receptor-positive/HER2− metastatic breast cancer who have progressed after pretreatment with CDK4/6 inhibitors (13).

So far, many clinical trials were designed to compare the efficacy and safety between CDK4/6 inhibitors or PI3K/AKT/mTOR inhibitors and endocrine monotherapy. Nevertheless, there were no direct comparisons between the two categories of inhibitors until now. Our previous network meta-analysis was conducted to work out this problem (14), after which a large number of randomized controlled trials (RCTs) emerged. Therefore, we performed this updated systematic review and network meta-analysis, attempting to replenish the latest survival outcomes and incorporate all the eligible studies.

## Methods

This study was performed according to the Preferred Reporting Items for Systematic Reviews and Meta-analyses–Network Meta-Analyses (PRISMA-NMA) checklist (15). The study protocol was consistent with that of one previous systematic review and network meta-analysis conducted by our research group (14). This analysis was registered in PROSPERO (https://www.crd.york.ac.uk/PROSPERO/) with the registration number CRD42022321172.

### Search strategy and selection criteria

Two independent authors (Xu and Wang) searched the literature from PubMed, Embase, the Cochrane Library, and ClinicalTrials.gov from 1 February 2020 since the cutoff date of the original article was 31 January 2020, with our last search on 23 November 2021. In addition, the annual conferences of American Society of Clinical Oncology, European Society of Medical Oncology, San Antonio Breast Cancer Symposium, and Chinese Society of Clinical Oncology were replenished for integrity. The main search strings that we used were as follows: “breast cancer”, “HER2”, “hormone receptor”, “metastasis”, “CDK4/6 inhibitors”, “PI3K inhibitors”, “AKT inhibitors”, “mTOR inhibitors”, and “endocrine therapy”, among which diverse concrete agents were searched. The key terms and free terms were combined in every possible form. Full searching strategy was detailed in **Supplementary Table 1**. The selection criteria were as previously displayed (14). Phase 2/3 RCTs meeting the following criteria were included (1): involving adults with hormone receptor-positive and HER2-negative metastatic breast cancer (2); participants were treated with regimens containing CDK4/6 inhibitors or PI3K/AKT/mTOR inhibitors; and (3) data regarding survival were available. Studies portrayed as single-arm trials or retrospective analyses, chemotherapy-containing regimens, positive or ambiguous HER2 status, and incomplete survival or follow-up data were excluded.

### Data extraction and quality assessment

On the basis of the designed protocol, baseline characteristics including clinical trial name, first author, published time, study phase, trial design, number of participants, lines of previous therapy and metastatic sites were extracted from each RCT. The progression-free survival (PFS) and overall survival (OS) were primary endpoints, whereas treatment-related adverse events (TRAEs) based on the Common Toxicity Criteria for Adverse Events (CTCAE) version 5.0 were the second, which consisted of not only grades 3–5 similar to the original article but also all-grade TRAE data. Other outcomes including time to the first chemotherapy and PFS of different metastatic site subgroups were also collected.

For every included study, the potential risk of bias was assessed by Cochrane Collaboration’s tool (16), including six pre-specified domains: selection bias, performance bias, detection bias, attrition bias, reporting bias, and other bias. All studies were evaluated as high, low, or unclear risk according to the documented methodological quality. Review Manager (version 5.3, Nordic Cochrane Centre) was employed to assess the risk of bias. Two investigators (Xu and Wang) independently contributed to the above process. Discussion was required with a third reviewer (Han) when disagreement existed.

### Data synthesis and statistical analysis

This network meta-analysis was performed by R software (version 4.1.1) with “gemtc” package based on Bayesian random effects models. Regarding survival data, hazard ratios (HRs) and 95% confidence intervals (CIs) extracted from each RCT were used to generate mean log HRs and according standard errors that were required for the subsequent analysis. The formulae applied were reported previously by Woods et al. (17). Regarding categorical data, summary odds ratios (ORs) with 95% credible intervals (CrIs) were estimated. Because not all the included studies reported all the endpoints of interest, each individual network plot of different outcomes was generated. In the network plot, each node represented an intervention and every two different nodes were connected with a single line if direct comparisons existed. The studies comparing different agents of the same classification were excluded. Markov chain Monte Carlo (MCMC) approach was utilized to build the network meta-analysis. In brief, the amount of adaptation and simulation iterations was 10,000 and 50,000, respectively, with the thinning interval set as 10. The surface under the cumulative ranking curve (SUCRA) was utilized to assess the relative ranking probabilities of different treatments for each outcome. The interventions were ordered by the SUCRA percentage (range, 0%–100%). A two-sided P-value below 0.05 was regarded as statistically significant.

## Results

### Study characteristics

**Figure 1:**
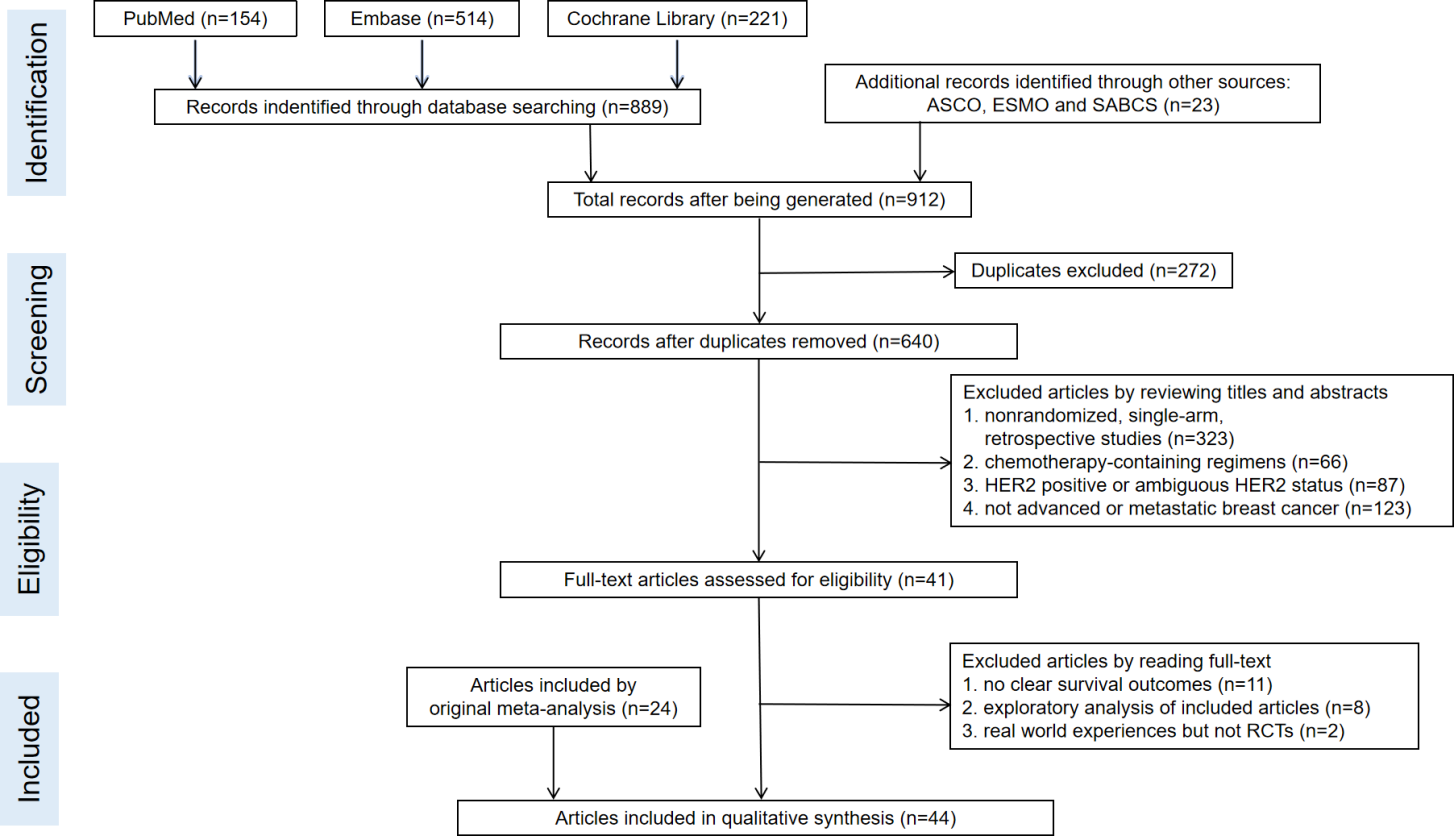
The flow chart of detailed literature screening process.

**Figure 2:**
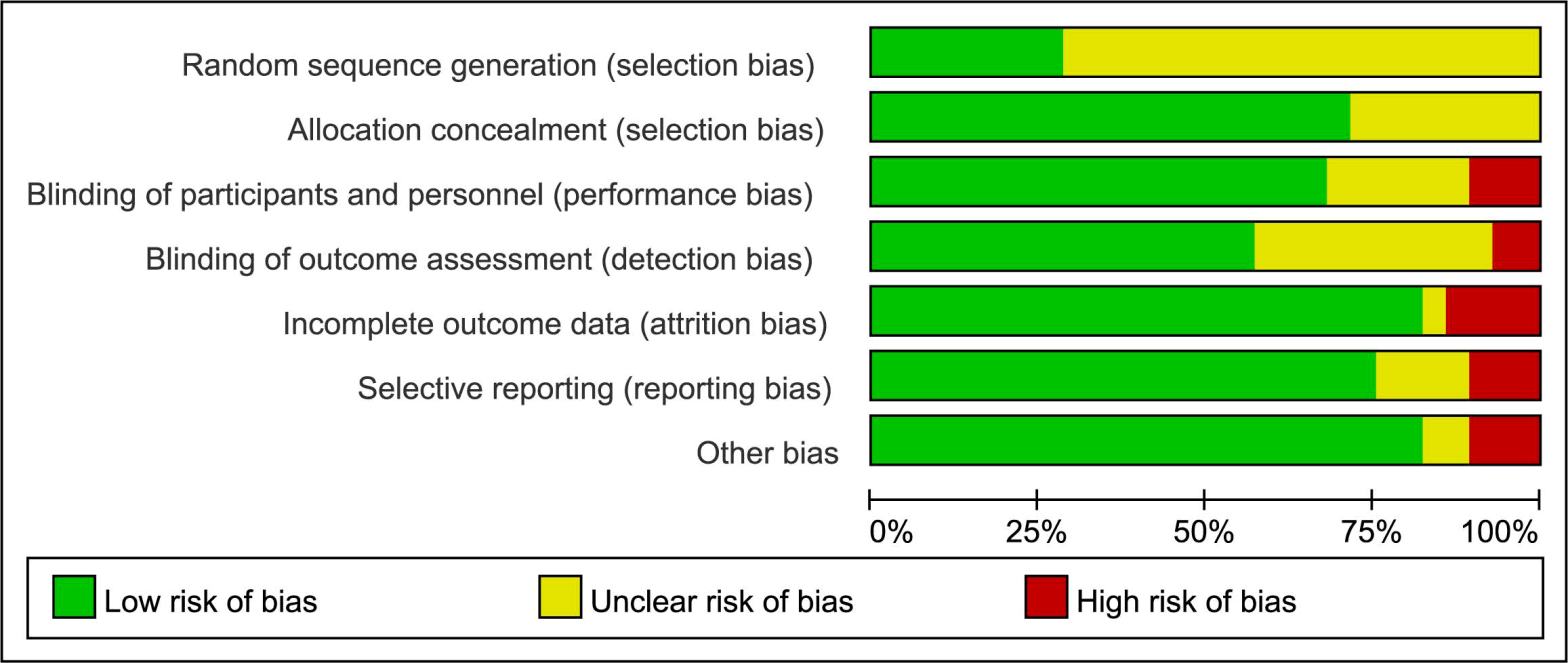
Risk of bias summary.

A total of 28 RCTs were included in our study, of which 20 RCTs had been included in the previous review and eight RCTs were newly subsumed. The detailed process of literature screening was shown in **Figure 1**. Because this was an updated analysis of previous research (14), we mainly retrieved the newly published articles after 31 January 2020. Meanwhile, we also updated the OS and other data of the original 20 RCTs. The quality assessment of all included studies was illustrated in **Figure 2**. In total, 12,129 patients with hormone receptor-positive and HER2-negative advanced breast cancer were included, among which 7,193 and 4,936 patients were in the experimental and control groups, respectively. The basic characteristics of enrolled studies including author, published time, study phase, interventions, the prior treatment lines, and different metastatic sites were listed in **Table 1**. The study was dissected into different cohorts if it consisted more than one intervention comparison. **Table 2** recorded PFS, OS, time to the first chemotherapy of all enrolled populations, and PFS of different subgroups.

**Table 1:** Baseline characteristics of 28 RCTs.

Study	Author. Published Time	Phase	Regimen (No. of Patients)		Prior Lines of Therapy (%)		Bone-Only metastasis (%)		Visceral metastasis (%)	
			I-group	C-group	I-group	C-group	I-group	C-group	I-group	C-group
PALOMA-1/ TRIO-18 (18, 19)	Richard S. Finn et al. 2015.01/2020.07	II	Pabociclib+ Letrozole (84)	Letrozole (81)	0(100)	0(100)	19	15	45	53
PALOMA-2 (20)	Richard S Finn et al. 2016.11	III	Pabociclib+ Letrozole (444)	Letrozole (222)	0(100)	0(100)	23.2	21.6	48.2	49.5
PALOMA-3 (21–23)	Robert H Lurie et al., 2015.07/2018.10/2021.06	III	Pabociclib+ Fulvestrant (347)	Placebo+ Fulvestrant (174)	0(21)/1(41)/ 2(37)/≥3(11)	0(23)/1(48)/ 2(21)/≥3(7)	NA	NA	59.4	60.3
PALOMA-4 (24)	Binghe Xu et al. 2021.08	III	Pabociclib+ Letrozole (169)	Placebo+ Letrozole (171)	0(100)	0(100)	NA	NA	55.6	56.1
PARSIFAL (25)	Antonio L Cussac,et al. 2021.08	II	Palbociclib+ Fulvestrant (243)	Palbociclib+ Letrozole (243)	0(100)	0(100)	NA	NA	47.3	48.6
FLIPPER (26)	J Albanell et al. 2021.12	II	Palbociclib+ Fulvestrant (94)	Placebo+ Fulvestrant (95)	0(100)	0(100)	NA	NA	60.6	60
MONALEESA-2 (27–29)	G N Hortobagyi et al. 2016.11/2018.07/2021.08	III	Ribociclib+ Letrozole (334)	Placebo+ Letrozole (334)	0(100)	0(100)	20.7	23.4	59	58.7
MONALEESA-3 (30–32)	Dennis J. Slamon et al., 2018.08/2020.02/2021.08	III	Ribociclib+ Fulvestrant (484)	Placebo+ Fulvestrant (242)	0(49.2)/ 1(48.8)	0(53.3)/1(48.0)	21.3	21.1	60.5	60.3
MONALEESA-7 (33, 34)	Debu Tripathy et al. 2018.05/2019.06	III	Ribociclib + TAM/NSAI (335)	Placebo + TAM/NSAI (337)	0(100)	0(100)	24	23	58	56
MONARCH-2 (35, 36)	George W Sledge Jr, et al. 2017.09/2020.01	III	Abemaciclib + Fulvestrant (446)	Placebo + Fulvestrant (223)	1(100)	1(100)	27.6	25.6	54.9	57.4
MONARCH-3 (37–39)	Stephen Johnston et al., 2017.11/2019.01/2021.06	III	Abemaciclib + NSAI (328)	Placebo + NSAI (165)	0(100)	0(100)	21.3	23.6	52.4	53.9
MONARCH plus (a) (40)	Qingyuan Zhang et al. 2020/08	III	Abemaciclib + NSAI (207)	Placebo + NSAI (99)	0(100)	0(100)	NA	NA	60.9	59.6
MONARCH plus (b) (40)	Qingyuan Zhang et al. 2020/08	III	Abemaciclib + Fulvestrant (104)	Placebo + Fulvestrant (53)	1(100)	1(100)	NA	NA	61.5	58.5
next MONARCH(a) (41)	Erika Hamilton et al. 2021.06	II	Abemaciclib 150 mg + Tamoxifen (78)	Abemaciclib 150 mg (79)	NA	NA	NA	NA	61.5	62
next MONARC(b) (41)	Erika Hamilton et al. 2021.06	II	Abemaciclib 150 mg (79)	Abemaciclib200 mg + loperamide (77)	NA	NA	NA	NA	62	62.3
DAWNA-1 (42)	Binghe Xu et al. 2021.06	III	Dalpiciclib + Fulvestrant (241)	Placebo + Fulvestrant (120)	1(72.6)/2(27.4)	1(72.5)/2(27.5)	17.4	15.8	58.9	62.5
SOLAR-1 (a) (43, 44)	F. André et al. 2019.05/2021.02	III	Alpelisib + Fulvestrant (169)	Placebo + Fulvestrant (172)	0(52.1)/1(46.7)	0(51.7)/1(47.7)	24.9	20.3	55	58.1
SOLAR-1 (b) (43, 44)	F. André et al. 2019.05/2021.03	III	Alpelisib + Fulvestrant (115)	Placebo + Fulvestrant (116)	0(61.7)/1(36.5)	0(53.4)/1(45.7)	22.6	19.8	57.4	63.8
Yen-Shen Lu, et al (45)	Yen-Shen Lu et al. 2021.01	Ib	Alpelisib + Tamoxifen + Goserelin (16)	Buparlisib + Tamoxifen + Goserelin(13)	0(100)	0(100)	33.3	7.7	NA	NA
BELLE-2 (46, 47)	José Baselga et al. 2017.07/2018.11	II	Buparlisib + Fulvestrant (576)	Placebo + Fulvestrant (571)	0(27)/1(53)/ ≥2(19)	0(25)/1(53)/ ≥2(22)	NA	NA	59	59
BELLE-3 (48)	Angelo Di Leo et al. 2018.01	III	Buparlisib + Fulvestrant (289)	Placebo + Fulvestrant (143)	1(30)/2(57)/≥3(13)	1(34)/2(53)/ ≥3(13)	15	13	73	72
SANDPIPER(a) (49)	S Dent et al. 2021.02	III	Taselisib + Fulvestrant (417)	Placebo + Fulvestrant (214)	NA	NA	NA	NA	NA	NA
SANDPIPER(b) (49)	S Dent et al. 2021.02	III	Taselisib + Fulvestrant (340)	Placebo + Fulvestrant (176)	NA	NA	20.6	18.2	59.1	58.5
POSEIDON (50)	M Oliveira et al. 2021.09	II	Taselisib + Tamoxifen (76)	Placebo + Tamoxifen (76)	0(64)/ 1(36)	0(67)/1(33)	NA	NA	NA	NA
FERGI(a) (51)	Ian E Krop et al. 2016.06	II	Pictilisib + Fulvestrant (89)	Placebo + Fulvestrant (79)	0(27)/1(37)/ 2 (26)/≥3(10)	0(25)/1(46)/ 2(19)/≥3(10)	21	22	57	53
FERGI(b) (51)	Ian E Krop et al. 2016.06	II	Pictilisib + Fulvestrant (41)	Placebo + Fulvestrant (20)	0(12)/1(27)/ 2(20)/≥3(41)	0(10)/1(35)/ 2(25)/≥3(30)	17	25	51	50
FAKTION (52)	Robert H Jones et al. 2020.03	II	Capivasertib + Fulvestrant (69)	Placebo + Fulvestrant (71)	0(13)/1(57)/ ≥2(29)	0(8)/1(63)/ ≥2(28)	14	11	71	66
BOLERO-2 (53, 54)	J Baselga et al., 2011.12 M. Piccart, et al., 2014.09	III	Everolimus + Exemestane (485)	Exemestane (239)	1(16)/2(30)/ ≥3(54)	1(18)/2(30)/ ≥3(53)	NA	NA	56	56
TAMRAD (55)	T Bachelot et al. 2021.08	II	Everolimus + Tamoxifen (54)	Tamoxifen (57)	NA	NA	25	30	49	57
MANTA(a) (56)	Peter Schmid et al. 2019.11	II	Visusertib + Fulvestrant (101)	Fulvestrant (66)	0(44)/1(45)/ ≥2(12)	0(44)/1(41)/ ≥2(15)	24	27	63	62
MANTA(b) (56)	Peter Schmid et al. 2019.11	II	Visusertib + Fulvestrant (95)	Fulvestrant (66)	0(47)/1(38)/ ≥2(15)	0(44)/1(41)/ ≥2(15)	22	27	56	62
MANTA(c) (56)	Peter Schmid et al. 2019.11	II	Everolimus + Fulvestrant (64)	Fulvestrant (66)	0(42)/1(39)/ ≥2(19)	0(44)/1(41)/ ≥2(15)	17	27	69	62
PrE0102 (57)	Noah Kornblum et al. 2018.06	II	Everolimus + Fulvestrant (66)	Placebo + Fulvestrant (65)	NA	NA	NA	NA	NA	NA
MIRACLE (58)	Ying Fan et al. 2021.08	II	Everolimus + Letrozole + OFS (101)	Letrozole + OFS (98)	0(100)	0(100)	NA	NA	57.4	58.2
LEO (59, 60)	Jae Ho Jeong et al. 2020.12/2021.04	II	Everolimus + Leuprorelin + Letrozole (92)	Leuprorelin + Letrozole (45)	0(49)/1(34)/≥2(17)	0(58)/1(29)/ ≥2(13)	5.4	13.3	60.9	60

Six RCTs were dissected into different cohorts, wherein SOLAR-1, SANDPIPER, and FERGI were divided according to PIK3CA mutation status, nextMONARCH and MANTA compared different usage and dosage of one specific drug, MONARCH plus was separated by different endocrine agents (NSAI or fulvestrant). RCTs, randomized clinical trials; NA, not available; TAM, tamoxifen; NSAI, non-steroidal aromatase inhibitors; I-group, interventional group; C-group, control group.

**Table 2:** Survival outcomes of RCTs.

Study	Median PFS (HR,95% CI)	Median OS (HR,95% CI)	Time to First Chemotherapy (HR,95% CI)	Visceral Metastasis (HR,95% CI)	Non-Visceral Metastasis (HR,95% CI)	Bone-Only Metastasis (HR,95% CI)	Liver Metastasis (HR,95% CI)
PALOMA-1/ TRIO-18 (18, 19)	20.2 m *vs*. 10.2 m (0.488, 0.319–0.748)	37.5 m *vs*. 34.5 m (0.897, 0.623–1.294)	0.662 (0.445–0.989)	NA	NA	NA	NA
PALOMA-2 (20)	24.8 m *vs*. 14.5 m (0.58, 0.46–0.72)	NA	NA	0.6 (0.47–0.85)	0.50 (0.36–0.70)	0.36 (0.22–0.59)	NA
PALOMA-3 (21–23)	9.5 m *vs*. 4.6 m (0.46, 0.36–0.59)	34.8 m *vs*. 28.0 m (0.81, 0.65–0.99)	NA	0.45 (0.32–0.63)	0.36 (0.22–0.60)	NA	NA
PALOMA-4 (24)	21.5 m *vs*. 13.9 m (0.68, 0.53–0.87)	NA	NA	0.657 (0.467–0.925)	0.700 (0.488–1.002)	NA	NA
PARSIFAL (25)	27.9 m *vs*. 32.8 m (1.13, 0.89–1.45)	NA *vs*. NA (1.00, 0.68–1.48)	NA	1.27 (0.91–1.77)	0.97 (0.67–1.40)	NA	NA
FLIPPER (26)	31.8 m *vs*. 22.0 m (0.48, 0.37–0.64)	NA	NA	0.45 (0.32–0.63)	0.62 (0.39–0.97)	1.13 (0.53–2.41)	0.56 (0.32–0.99)
MONALEESA-2 (27–29)	25.3 m *vs*. 16.0 m (0.568, 0.457–0.704)	63.9 m *vs*. 51.4 m (0.76, 0.63–0.93)	0.74 (0.61–0.91)	NA	NA	0.642 (0.393–1.048)	NA
MONALEESA-3 (30–32)	37.4 m *vs*. 28.1 m (0.693, 0.57–0.844)	53.7 m *vs*. 41.5 m (0.73, 0.59–0.90)	0.704 (0.566–0.876)	0.804 (0.596–1.083)	NA	NA	NA
MONALEESA-7 (33, 34)	23.8 m *vs*. 13.0 m (0.55, 0.44–0.69)	58.7 m *vs*. 48.0 m (0.763, 0.608–0.956)	0.694 (0.556–0.867)	0.698 (0.462–1.054)	NA	0.70 (0.41–1.19)	NA
MONARCH-2 (35, 36)	16.9 m *vs*. 9.3 m (0.536, 0.445–0.645)	46.7 m *vs*. 37.3 m (0.757, 0.606–0.945)	0.625 (0.501–0.779)	0.471 (0.371–0.598)	NA	0.580 (0.398–0.844)	NA
MONARCH-3 (37–39)	28.2 m *vs*. 14.8 m (0.525, 0.415–0.665)	NA	0.513 (0.380–0.691)	0.567 (0.407–0.789)	NA	0.471 (0.280–0.793)	0.449 (0.259–0.777)
MONARCH plus (a) (40)	NA *vs*. 14.7 m (0.499, 0.346–0.719)	NA	NA	0.615 (0.396–0.955)	0.335 (0.175–0.639)	NA	0.385 (0.194–0.763)
MONARCH plus (b) (40)	11.5 m *vs*. 5.6 m (0.376, 0.240–0.588)	NA	NA	0.423 (0.247–0.724)	0.328 (0.149–0.722)	NA	0.513 (0.270–0.974)
next MONARCH(a) (41)	9.1 m *vs*. 6.5 m (0.805, 0.551–1.177)	24.2 m *vs*. 20.8 m (0.620, 0.397–0.969)	NA	NA	NA	NA	NA
next MONARC(b) (41)	6.5 m *vs*. 7.4 m (1.045, 0.711–1.535)	20.8 m *vs*. 17.0 m (0.956, 0.635–1.438)	NA	NA	NA	NA	NA
DAWNA-1 (42)	13.6 m *vs*. 7.7 m (0.45, 0.32–0.64)	NA	0.47 (0.32–0.69)	0.48 (0.33–0.70)	0.36 (0.20–0.63)	0.76 (0.31–1.85)	NA
SOLAR-1 (a) (43, 44)	11.0 m *vs*. 5.7 m (0.65, 0.50–0.85)	39.3 m *vs*. 31.4 m (0.86, 0.64–1.15)	0.72 (0.54–0.95)	NA	NA	NA	NA
SOLAR-1 (b) (43, 44)	7.4 m *vs*. 5.6 m (0.85, 0.58–1.85)	NA	NA	NA	NA	NA	NA
Yen-Shen Lu, et al (45)	25.2 m *vs*. 20.6 m (NA, NA–NA)	NA	NA	NA	NA	NA	NA
BELLE-2 (46, 47)	6.9 m *vs*. 5.0 m (0.78, 0.67–0.89)	33.2 m *vs*. 30.4 m (0.87, 0.74–1.02)	NA	0.76 (0.62–0.92)	0.79 (0.58–1.07)	0.66 (0.46–0.95)	NA
BELLE-3 (48)	3.9 m *vs*. 1.8 m (0.67, 0.53–0.84)	NA	NA	0.56 (0.43–0.74)	0.96 (0.61–1.50)	1.06 (0.52–2.15)	NA
SANDPIPER(a) (49)	NA	NA	NA	NA	NA	NA	NA
SANDPIPER(b) (49)	9.0 m *vs*. 5.4 m (0.66, 0.51–0.86)	NA	NA	0.74 (0.56–1.00)	0.72 (0.49–1.04)	0.58 (0.33–1.01)	0.73 (0.51–1.04)
POSEIDON (50)	4.8 m *vs*. 3.2 m (0.63, 0.43–0.93)	20.9 m *vs*. 24.4 m (0.97, 0.63–1.5)	NA	NA	NA	NA	NA
FERGI(a) (51)	6.6 m *vs*. 5.1 m (0.74, 0.52–1.06)	NA	NA	0.74 (0.46–1.18)	0.70 (0.41–1.27)	0.57 (0.32–1.02)	NA
FERGI(b) (51)	5.4 m *vs*. 10.0 m (1.07, 0.53–2.18)	NA	NA	NA	NA	NA	NA
FAKTION (52)	10.3 m *vs*. 4.8 m (0.58, 0.39–0.84)	26.0 m *vs*. 20.0 m (0.59, 0.34–1.05)	NA	NA	NA	NA	NA
BOLERO-2 (53, 54)	10.6 m *vs*. 4.1 m (0.36, 0.27–0.47)	31.0 m *vs*. 26.6 m (0.89, 0.73–1.10)	NA	0.47 (0.37–0.80)	0.41 (0.31–0.55)	NA	NA
TAMRAD (55)	8.6 m *vs*. 4.5 m (0.54, 0.36–0.81)	NA *vs*. 32.9 m (0.45, 0.24–0.81)	NA	NA	NA	NA	NA
MANTA(a) (56)	7.6 m *vs*. 5.4 m (0.88, 0.63–1.24)	27.1 m *vs*. 24.4 m (NA, NA–NA)	NA	NA	NA	NA	NA
MANTA(b) (56)	8.0 m *vs*. 5.4 m (0.79, 0.55–1.12)	24.2 m *vs*. 24.4 m (NA, NA–NA)	NA	NA	NA	NA	NA
MANTA(c) (56)	12.3 m *vs*. 5.4 m (0.63, 0.42–0.92)	NA *vs*. 24.4 m (NA, NA–NA)	NA	NA	NA	NA	NA
PrE0102 (57)	10.3 m *vs*. 5.1 m (0.61, 0.40–0.92)	31.4 m *vs*. 28.3 m (1.31, 0.72–2.38)	NA	NA	NA	NA	NA
MIRACLE (58)	19.4 m *vs*. 12.9 m (0.64, 0.46–0.89)	NA	NA	0.762 (0.503–1.157)	0.469 (0.270–0.817)	NA	NA
LEO (59, 60)	18.1 m *vs*. 13.8 m (0.73, 0.48–1.11)	48.3 m *vs*. 50.8 m (NA, NA–NA)	NA	0.58 (0.34–0.99)	1.09 (0.53–2.21)	NA	NA

PFS, progression-free survival; OS, overall survival; HR, hazard ratio; CI, confidence interval; m, months; NA, not available.

**Figure 3:**
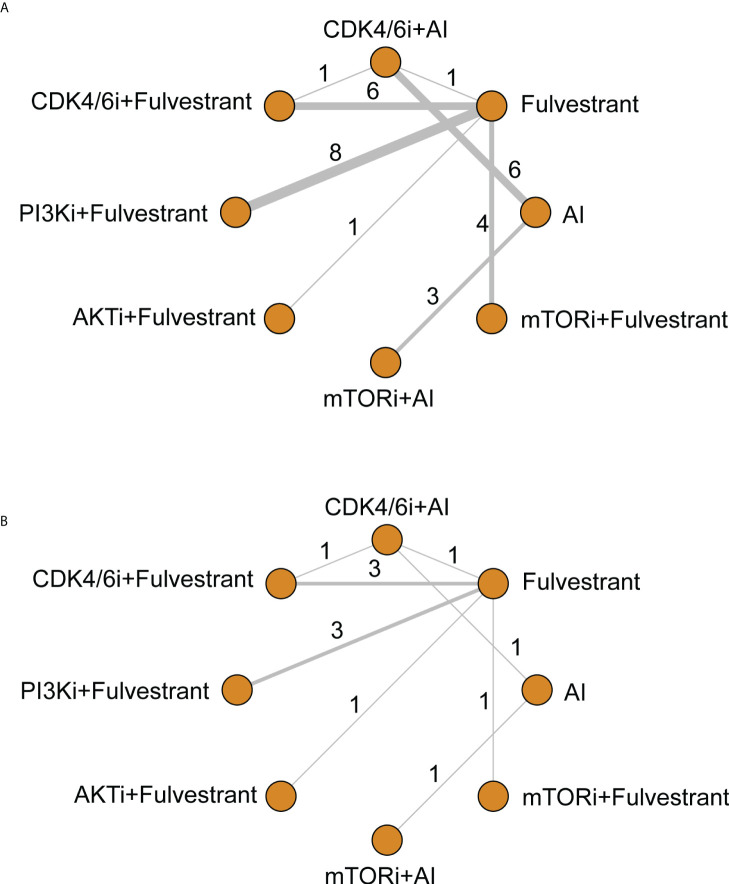
Network of comparative interventions for **(A)** PFS and **(B)** OS. CDK4/6i, CDK4/6 inhibitors; PI3K, PI3K inhibitors; AKTi, AKT inhibitors; mTORi, mTOR inhibitors; AI, aromatase inhibitors.

There were 13 different treatment regimens in the 28 RCTs, among which eight interventions were incorporated into the network meta-analysis (**Figure 3**). One of the interventions was excluded from the network because the corresponding RCT (45) conducted comparisons within PI3K inhibitors (alpelisib and buparlisib), without connections with other interventions in the network. The involved agents were CDK4/6 inhibitors (including palbociclib, ribociclib, abemaciclib, and dalpiciclib), PI3K inhibitors (including alpelisib, buparlisib, pictilisib, and taselisib), AKT inhibitor (capivasertib), and mTOR inhibitors (everolimus and vistusertib). All kinds of AIs, fulvestrant, and tamoxifen were amalgamated into ET in subsequent data analysis.

### Survival outcomes

Intervention arms that reported PFS or OS with available HRs and 95% CIs were utilized for data synthesis. Regarding PFS and OS, there were eight different interventions that formed a network, and the pairwise comparisons of them were shown in **Table 3**. In this table, the treatments were sequenced according to the SUCRA values of PFS (**Table 4A**), whereas the SUCRA values of OS were displayed in **Table 4B**. All varieties of CDK4/6 inhibitors and PI3K/AKT/mTOR inhibitors plus ET showed remarkable advantages in PFS compared with fulvestrant monotherapy, with HR and 95% CrI valued as 0.47 (0.32–0.69) for mTORi plus AI, 0.52 (0.40–0.66) for CDK4/6i plus AI, 0.53 (0.46–0.61) for CDK4/6i plus fulvestrant, 0.57 (0.36–0.90) for AKTi plus fulvestrant, 0.72 (0.63–0.83) for PI3Ki plus fulvestrant, and 0.73 (0.58–0.91) for mTORi plus fulvestrant. Compared with AI, significant improvements of PFS were observed in three therapeutic regimens, consisting of mTORi plus AI (HR, 0.51; 95% CrI, 0.41–0.66), CDK4/6i plus AI (HR, 0.56; 95% CrI, 0.48–0.66), and CDK4/6i plus fulvestrant (HR, 0.58; 95% CrI, 0.43–0.77). CDK4/6i plus ET saliently improved the PFS compared with PI3Ki plus fulvestrant (CDK4/6i plus AI: HR, 0.72; 95% CrI, 0.54–0.95; CDK4/6i plus fulvestrant: HR, 0.74; 95% CrI, 0.6–0.8) and mTORi plus fulvestrant (CDK4/6i plus AI: HR, 0.71; 95% CrI, 0.51–0.99; CDK4/6i plus fulvestrant: HR, 0.73; 95% CrI, 0.56–0.94). In addition, mTORi plus AI showed a better PFS than PI3Ki plus fulvestrant (HR, 0.66; 95% CrI, 0.44–0.98). With respect to OS, CDK4/6i plus either AI or fulvestrant significantly prolonged the survival compared with fulvestrant monotherapy (HR, 0.76; 95% CrI, 0.61–0.96; HR, 0.76; 95% CrI, 0.66–0.89, respectively). No significant difference was observed in OS when comparing PI3K/AKT/mTOR inhibitors plus ET with endocrine monotherapy. In general, compared with ET (AI or fulvestrant), forest plots indicated that CDK4/6i significantly prolonged PFS than PAM pathway inhibitors (HR, 0.81; 95% CrI, 0.69–0.94) (**Supplementary Figure 1A**). To be more precise, the diversity seemed to mainly exist between CDK4/6i and PI3Ki (HR, 1.3; 95% CrI, 1.1–1.6) (**Supplementary Figure 1B**). However, the similar difference was not displayed in OS (**Supplementary Figures 1C, D**).

**Table 3:** Pairwise comparisons of 8 interventions for PFS and OS (HR, 95% CrI).

							
**mTORi + AI**	1.00 (0.61, 1.63)	0.99 (0.57, 1.72)	1.25 (0.57, 2.80)	0.86 (0.49, 1.52)	0.58 (0.25, 1.33)	0.89 (0.69, 1.17)	0.76 (0.44, 1.31)
0.91 (0.69, 1.22)	**CDK4/6i + AI**	1.00 (0.78, 1.28)	1.26 (0.68, 2.44)	0.87 (0.65, 1.16)	0.58 (0.30, 1.14)	0.90 (0.60, 1.35)	0.76 (0.61, 0.96)
0.89 (0.62, 1.32)	0.98 (0.77, 1.26)	**CDK4/6i + Fulvestrant**	1.27 (0.70, 2.39)	0.87 (0.69, 1.10)	0.58 (0.30, 1.13)	0.90 (0.56, 1.46)	0.76 (0.66, 0.89)
0.83 (0.46, 1.50)	0.91 (0.54, 1.53)	0.93 (0.57, 1.48)	**AKTi +** **Fulvestrant**	0.69 (0.36, 1.26)	0.46 (0.19, 1.09)	0.71 (0.33, 1.50)	0.60 (0.33, 1.08)
**0.66** **(0.44, 0.98)**	**0.72** **(0.54, 0.95)**	**0.74** **(0.60, 0.89)**	0.79 (0.50, 1.27)	**PI3Ki +** **Fulvestrant**	0.67 (0.35, 1.31)	1.04 (0.63, 1.72)	0.88 (0.74, 1.05)
0.65 (0.42, 1.01)	**0.71** **(0.51, 0.99)**	**0.73** **(0.56, 0.94)**	0.78 (0.47, 1.30)	0.99 (0.76, 1.29)	**mTORi +** **Fulvestrant**	1.55 (0.71, 3.36)	1.32 (0.69, 2.48)
**0.51** **(0.41, 0.66)**	**0.56** **(0.48, 0.66)**	**0.58** **(0.43, 0.77)**	0.62 (0.36, 1.06)	0.78 (0.57, 1.08)	0.79 (0.55, 1.15)	**AI**	0.85 (0.53, 1.35)
**0.47** **(0.32, 0.69)**	**0.52** **(0.40, 0.66)**	**0.53** **(0.46, 0.61)**	**0.57** **(0.36, 0.90)**	**0.72** **(0.63, 0.83)**	**0.73** **(0.58, 0.91)**	0.92 (0.69, 1.22)	**Fulvestrant**

Contrast of PFS (on the lower triangle) and OS (on the upper triangle). The HRs lower than 1 revealed the favorable tendency of column-defining regimens for PFS and row-defining regimens for OS. Significant differences were bolded. HR, hazard ratio; CrI, credible interval; CDK4/6i, CDK4/6 inhibitors; PI3Ki, PI3K inhibitors; AKTi, AKT inhibitors; mTORi, mTOR inhibitors; AI, aromatase inhibitors.

**Table 4:** SUCRA values of each combination regimen for PFS (A) and OS (B).

(A) SUCRA values for PFS	
Interventions	SUCRA%
mTORi + AI	88.62
CDK4/6i + AI	77.94
CDK4/6i + Fulvestrant	75.5
AKTi + Fulvestrant	65.66
PI3Ki + Fulvestrant	38.18
mTORi + Fulvestrant	36.75
AI	13.39
Fulvestrant	3.96
(B) SUCRA values for OS	
Interventions	SUCRA%
AKTi + Fulvestrant	85.26
CDK4/6i + Fulvestrant	67.90
CDK4/6i + AI	67.19
mTORi + AI	64.18
AI	44.65
PI3Ki + Fulvestrant	41.99
Fulvestrant	19.15
mTORi + Fulvestrant	9.67

(SUCRA, surface under the cumulative ranking curve).

### Subgroup survival analysis

We then stratified the survival data based on prior treatment lines and metastatic sites. Pooled results suggested that there was no significant difference between CDK4/6 inhibitors and PI3K/AKT/mTOR inhibitors regarding PFS and OS in both first and second lines. HRs and 95% CrIs between CDK4/6 inhibitors and PI3K/AKT/mTOR inhibitors for first-line PFS, second-line PFS, first-line OS, and second-line OS were 1.2 (0.94–1.6), 1.1 (0.87–1.5), 1.0 (0.61–1.8), and 1.1 (0.54–2.0), respectively (**Supplementary Figure 2**). Subgroups with different metastatic sites were classified as visceral (generally defined as all lesions except breast, skin, soft tissue, lymph node, and bone), bone, and liver metastasis, of which PFS data were collected. In both visceral and non-visceral metastasis subgroups, PI3K inhibitors showed worse curative effects than CDK4/6 inhibitors (HR, 1.3: 95% CrI, 1.1–1.7; HR, 1.6; 95% CrI, 1.1–2.5, respectively) (**Supplementary Figure 3**). However, we did not discover conspicuous discrepancy in bone and liver metastatic subgroups (**Supplementary Figures 4**, **5**). In addition, there was no significant difference between PI3K inhibitors and CDK4/6 inhibitors in the time to the first subsequent chemotherapy (**Supplementary Figure 6**).

### Safety

Four kinds of hematological and 13 kinds of non-hematological TRAEs were collected, and they were divided into all grades and grade ≥ 3.

In terms of adverse effects within CDK4/6 inhibitors, pooled analysis demonstrated that abemaciclib was significantly better than others regarding neutropenia of grade ≥3 (OR, 0.035; 95% CrI, 0.0058–0.15) (**Supplementary Figure 7**). There was no significant difference in other hematological adverse events among groups of CDK4/6 inhibitors, regardless of all grades or grade ≥3. Among PI3K/AKT/mTOR inhibitors, both alpelisib (OR, 0.24; 95% CrI, 0.035–1.0) and burparlisib (OR, 0.27; 95% CrI, 0.052–0.96) tended to exhibit lower risk of all-grade stomatitis than everolimus (**Supplementary Figure 8**). Compared with capivasertib, vistusertib was more prone to bring nausea (OR, 6.6; 95% CrI, 1.6–27) (**Supplementary Figure 9**); alpelisib (OR, 3.1; 95% CrI, 1.2–8.3) and vistusertib (OR, 3.6; 95% CrI, 1.1–12) had significantly higher risk than pictilisib regarding anorexia (**Supplementary Figure 10**).

As for common safety concerns, CDK4/6 inhibitors and PI3K/AKT/mTOR inhibitors showed different profiles in hepatotoxicity, gastrointestinal (GI) toxicity, and hyperglycemia. Burparlisib and everolimus remarkably elevated alanine aminotransferase (ALT) concentration (OR, 7.0; 95% CrI, 2.8–16.0; OR, 4.1; 95% CrI, 1.5–13.0, respectively) in all grades compared with palbociclib (**Supplementary Figure 11**). Burparlisib showed similar tendency in aspartate aminotransferase (AST) elevation (**Supplementary Figure 12**). In addition, although not reaching statistical significance, dalpiciclib tended to have less hepatotoxicity (OR, 0.37; 95% CrI, 0.13–1.00; OR, 0.40; 95% CrI, 0.14–1.10, respectively) (**Supplementary Figures 11**, **12**). From **Supplementary Figure 13**, we observed that the risk of diarrhea of all grades caused by PI3K/AKT/mTOR inhibitors was significantly higher than that of palbociclib and ribociclib. Compared with palbociclib, all interventions except burparlisib and everolimus significantly increased the risk of diarrhea. Regarding hyperglycemia, compared with CDK4/6 inhibitors, three of four PI3K inhibitors were more likely to induce hyperglycemia of all grades, including alpelisib (OR, 11.0; 95% CrI, 2.3–50.0), burparlisib (OR, 10.0; 95% CrI, 2.9–48.0), and taselisib (OR, 6.5; 95% CrI, 1.6–34.0), whereas pictilisib, capivasertib, and everolimus showed the same trend without salient discrepancy (**Supplementary Figure 14**).

## Discussion

Although the long-term prognosis is favorable for patients with early breast cancer (61), the 5-year survival rate for patients whose disease has progressed or metastasized is still not optimistic (62). For hormone receptor+/HER2− subtype, not all patients are responsive to the first-line ET, and drug resistance and subsequent disease progression may eventually occur in some patients. Many inhibitors involved in the CDK4/6 and PAM signaling pathways are investigated, with great clinical efficacy and acceptable toxicities, confirmed by numerous RCTs (62). Despite the increasing use of these two kinds of agents in clinical practice, there is controversy about their advantages and disadvantages regarding efficacy and safety. Our original meta-analysis was the first pooling analysis that synthesized outcomes of many clinical trials and used indirect comparisons to shed light on the above issue (14). Considering that data of some RCTs continue to mature and many novel studies rise, we updated the previous meta-analysis. Compared with our previous research, the present study integrated much more data regarding both efficacy and toxicity. We also further explored the data of more subgroups and other endpoints.

The survival outcomes of our study showed that compared with endocrine monotherapy, both CDK4/6 inhibitors and PI3K/AKT/mTOR inhibitors acquired longer PFS and OS, demonstrated by the SUCRA values. Favorable PFS reached statistically significance nearly in all the combination regimens compared with monotherapy except for PI3K/AKT/mTOR inhibitors plus fulvestrant versus AI. As for the pairwise comparisons between different targeted therapeutic groups, CDK4/6 inhibitors took conspicuous superiority than PI3K/AKT/mTOR inhibitors in short-term PFS but not long-term OS, and the difference might mainly exist between CDK4/6 and PI3K inhibitors. Notably, although mTOR inhibitors plus AI ranked the highest from SUCRA values of PFS, pairwise comparisons did not indicate this treatment strategy was better than CDK4/6 inhibitors plus ET. Our study only suggested the tendency of CDK4/6 inhibitor-containing treatment regimens toward better OS from the view of SUCRA values. Currently, there are still challenges in differentiating the population that can acquire OS benefit from CDK4/6 inhibitors due to the profound heterogeneity of patients (63).

Considering that the timing of treatment with PI3K/AKT/mTOR inhibitors is generally later than CDK4/6 inhibitors, past studies were usually layered by treatment lines (64, 65). We collected information on the lines of previous ET and classified the survival outcomes accordingly to eliminate the potential bias. However, after analyzing the data by this way, we did not observe obvious advantages in PFS and OS of CDK4/6 inhibitors over PI3K and mTOR inhibitors in terms of different treatment lines, which was discordant with our original analysis (14). The discrepancy might be due to the fact that the delineation of the treatment lines in previous meta-analysis was not very refined and clear; meanwhile, the data of new clinical trials became available. Current study findings were also inconsistent with another meta-analysis conducted by Leung et al. that compared CDK4/6 and PI3K/AKT/mTOR inhibitors plus fulvestrant in the setting of second-line treatment, of which the synthetic outcomes supported the superior efficacy of CDK4/6 inhibitors (65). Some of the clinical trials included in the above meta-analysis did not contain patients of second line exclusively. Thus, the indistinct prior treatment lines may partly explain the different results between two meta-analyses because we divided the treatment lines more precisely. Nevertheless, we noted that the efficacy of CDK4/6 inhibitors was superior to other kinds of inhibitors both in first and second lines from the SUCRA values. Another point to be noted was that there only existed data of PI3K inhibitors regarding OS for first-line treatment. Therefore, we could not conclude that CDK4/6 inhibitors were superior to all PAM pathway inhibitors in this situation. In summary, we believe that the overall efficacy of PI3K/AKT/mTOR inhibitors is not inferior to that of CDK4/6 inhibitors, but only because their application is usually in later lines. With respect to the data stratified by metastatic sites and the time to first subsequent chemotherapy, salient advantages of CDK4/6 inhibitors only existed in the visceral and non-visceral subgroups.

As for the safety, CDK4/6 inhibitors and PI3K/AKT/mTOR inhibitors each had their own specific profiles, whereas there were also common concerns between the two. Among CDK4/6 inhibitors, previous research studies indicated that abemaciclib was associated with a low rate of neutropenia and a high incidence of GI toxicity (66, 67). Our present analysis confirmed the above findings. The hematologic toxicity of CDK4/6 inhibitors is mainly caused by the inhibiting effect on CDK6 in hematopoietic cells, whereas abemaciclib has a 14-fold higher affinity for CDK4 than CDK6 (68). Moreover, abemaciclib also exerts inhibiting effect on CDK9, which is considered to be related with the increased GI toxicity like diarrhea (69). Within PI3K/AKT/mTOR inhibitors, the incidence of stomatitis, nausea, anorexia, and hepatic toxicity of different agents showed significant differences. Therein, everolimus led to more stomatitis, vistusertib and alpelisib increased the risk of digestive disorders, whereas both burparlisib and everolimus remarkably elevated the ALT/AST levels according to our analysis. In the comparisons of the two categories, PI3K/AKT/mTOR inhibitors generally incurred more diarrhea than palbocilib and ribociclib. In addition, hyperglycemia is reported in nearly all kinds of PI3K/AKT/mTOR inhibitors but only in two CDK4/6 inhibitors (palbociclib and dalpiciclib). Pooled analysis revealed the tendency for PAM inhibitors especially PI3K inhibitors to result in escalated blood glucose level. Hyperglycemia is one of the common on-target side effects of PI3K inhibitors due to the dysregulation of glucose metabolism that warrants prevention, monitoring, and treatment (70, 71). Our original study mainly focused on the severe TRAEs of grade ≥3, whereas current study further supplemented the data regarding all grades and capivasertib-caused toxicity. On the basis of the above results, individualized treatment options could be deliberated.

Inevitably, there existed several limitations. On one hand, the enrolled patients were mainly postmenopausal, and the data for premenopausal women were still insufficient. However, this issue was hard to be fully addressed due to the restriction by the inclusion criteria of original trials. On the other, all data in our meta-analysis were extracted from published literatures without original prospective outcomes, which may cause bias to the present results. In addition, although we have updated the data, some interim results were still from conference abstracts without available full texts. In addition, the heterogeneity among included studies was inevitable although we performed subgroup analyses to minimize it. Nevertheless, the heterogeneity was low from I^2^ values, which indicated the satisfactory credibility of our study.

However, our investigation is of clinical significance to some extent and could provide clues for the future practice. Although PI3K/AKT/mTOR inhibitors are recommended for later lines of treatment than CDK4/6 inhibitors in hormone receptor+/HER2− metastatic breast cancer patients, the relative equivalent efficacy in different treatment lines provide more reasons for PI3K/AKT/mTOR inhibitors to be used in earlier clinical settings. At present, numerous studies have focused on this aspect and evaluate the efficacy of PI3K/AKT/mTOR inhibitors in the setting of neoadjuvant, adjuvant, and first-line treatments (72–75). Despite the similar efficacy of two kinds of agents, the safety profiles varied on the basis of the current results, which signified that different recommendations could be made to patients accordingly given the different tolerance to TRAEs. In addition, several *in vitro* and *in vivo* preclinical studies indicated that the triplet combination strategy of CDK4/6 and PI3K/AKT/mTOR inhibitors with traditional endocrine agents could overcome endocrine resistance and show synergistic effects (76–79). The endocrine resistance remains a difficult problem, of which the mechanism is complicated and not yet clearly defined (5). The combination therapy is expected to restrain and reverse drug resistance and tumor metastasis. As all mentioned above, this study provides further insight into CDK4/6 and PI3K/AKT/mTOR inhibitors, and we anticipate the implementation of large-scale RCTs with head-to-head comparisons to ultimately address the clinical issue.

## Conclusions

In conclusion, CDK4/6 inhibitors showed conspicuous superiority than PI3K/AKT/mTOR inhibitors regarding PFS, whereas the superiority no longer existed by balancing the treatment lines. Detailed subgroup analysis suggested the advantages of CDK4/6 inhibitors in the population with visceral and no-visceral metastatic sites. The safety profiles were diverse between two varieties of agents.

## Data availability statement

The original contributions presented in the study are included in the article/**Supplementary Material**. Further inquiries can be directed to the corresponding authors.

## Author contributions

HX, YaW, and YH: protocol development, data extraction, data analysis, and manuscript writing. YuW: data extraction and manuscript writing. JW and BX: protocol development and final manuscript review. All authors contributed to the article and approved the submitted version.

## Acknowledgments

We deeply appreciate all authors who performed and patients who participated in the included studies in our systematic review and meta-analysis.

## Conflict of interest

The authors declare that the research was conducted in the absence of any commercial or financial relationships that could be construed as a potential conflict of interest.

## Publisher’s note

All claims expressed in this article are solely those of the authors and do not necessarily represent those of their affiliated organizations, or those of the publisher, the editors and the reviewers. Any product that may be evaluated in this article, or claim that may be made by its manufacturer, is not guaranteed or endorsed by the publisher.

## Supplementary material

Supplementary Table 1 Full search strategy in **(A)** PubMed, **(B)** Embase and **(C)** the Cochrane Library.

Supplementary Figure 1 Overall comparisons between CKD4/6 inhibitors or PI3K/AKT/mTOR inhibitors and endocrine therapy for PFS **(A, B)** and OS **(C, D)**.

Supplementary Figure 2 Comparisons between CDK4/6 inhibitors and PI3K/AKT/mTOR inhibitors when stratified by treatment lines: **(A)** for first-line PFS, **(B)** for second-line PFS, **(C)** for first-line OS and **(D)** for second-line OS.

Supplementary Figure 3 PFS comparisons between CDK4/6 inhibitors and PI3K/AKT/mTOR inhibitors in visceral **(A)** and non-visceral **(B)** metastasis subgroups.

Supplementary Figure 4 PFS comparisons between CDK4/6 inhibitors and PI3K/AKT/mTOR inhibitors in bone-only metastasis subgroup.

Supplementary Figure 5 PFS comparisons between CDK4/6 inhibitors and PI3K/AKT/mTOR inhibitors in liver metastasis subgroup.

Supplementary Figure 6 Time to first subsequent chemotherapy comparisons between CDK4/6 inhibitors and PI3K inhibitors.

Supplementary Figure 7 ≥3 grade neutropenia comparisons within CDK4/6 inhibitors.

Supplementary Figure 8 All grade stomatitis comparisons within PI3K/AKT/mTOR inhibitors.

Supplementary Figure 9 All grade nausea comparisons within PI3K/AKT/mTOR inhibitors.

Supplementary Figure 10 All grade anorexia comparisons within PI3K/AKT/mTOR inhibitors.

Supplementary Figure 11 All grade elevated ALT concentrations comparison among all treatments.

Supplementary Figure 12 All grade elevated AST concentrations comparison among all treatments.

Supplementary Figure 13 All grade diarrhea comparisons among all treatments.

Supplementary Figure 14 All grade hyperglycemia comparisons among all treatments.
