# Supplementary material for: CDK4/6 inhibitors versus PI3K/AKT/mTOR inhibitors in women with hormone receptor-positive, HER2-negative metastatic breast cancer: An updated systematic review and network meta-analysis of 28 randomized controlled trials
Source: Front Oncol. 2022 Aug 24;12:956464. doi: 10.3389/fonc.2022.956464 (PMC9449843; doi:10.3389/fonc.2022.956464)

Supplementary Material

**Supplementary Table 1** Full search strategy in PubMed **(A)**, Embase **(B)** and the Cochrane Library **(C)**.

**Supplementary Figure 1** Overall comparisonss between CKD4/6 inhibitors or PI3K/AKT/mTOR inhibitors and endocrine therapy for PFS **(A and B)** and OS **(C and D)**.

**Supplementary Figure 2** Comparisonss between CDK4/6 inhibitors and PI3K/AKT/mTOR inhibitors when stratified by treatment lines: **(A)** for first line PFS, **(B)** for second line PFS, **(C)** for first line OS and **(D)** for second line OS.

**Supplementary Figure 3** PFS comparisonss between CDK4/6 inhibitors and PI3K/AKT/mTOR inhibitors in visceral **(A)** and non-visceral **(B)** metastasis subgroups.

**Supplementary Figure 4** PFS comparisons between CDK4/6 inhibitors and PI3K/AKT/mTOR inhibitors in bone-only metastasis subgroup.

**Supplementary Figure 5** PFS comparisons between CDK4/6 inhibitors and PI3K/AKT/mTOR inhibitors in liver metastasis subgroup.

**Supplementary Figure 6** Time to first subsequent chemotherapy comparisons between CDK4/6 inhibitors and PI3K inhibitors.

**Supplementary Figure 7** ≥3 grade neutropenia comparisons within CDK4/6 inhibitors.

**Supplementary Figure 8** All grade stomatitis comparisons within PI3K/AKT/mTOR inhibitors.

**Supplementary Figure 9** All grade nausea comparisons within PI3K/AKT/mTOR inhibitors.

**Supplementary Figure 10** All grade anorexia comparisons within PI3K/AKT/mTOR inhibitors.

**Supplementary Figure 11** All grade elevated ALT concentration comparisons among all treatments.

**Supplementary Figure 12** All grade elevated AST concentration comparisons among all treatments.

**Supplementary Figure 13** All grade diarrhea comparisons among all treatments.

**Supplementary Figure 14** All grade hyperglycemia comparisons among all treatments.

**Supplementary Table 1** Full search strategies.

1. Search strategy in PubMed, December 23^rd^, 2021.

| **Set** | **Search terms** | **Result** |
| --- | --- | --- |
| #1 | (breast[Title/Abstract] OR mammary[Title/Abstract]) AND (cancer[Title/Abstract] OR carcinoma[Title/Abstract] OR malignancy[Title/Abstract] OR neoplasm[Title/Abstract] OR tumor[Title/Abstract]) AND (HER-2[Title/Abstract] OR HER2[Title/Abstract] OR neu[Title/Abstract] OR ERBB2[Title/Abstract] OR human epidermal growth factor receptor 2[Title/Abstract]) AND (negative[Title/Abstract] OR low expressing[Title/Abstract] OR low expressed[Title/Abstract] OR –[Title/Abstract]) AND (hormone receptor[Title/Abstract] OR estrogen receptor[Title/Abstract] OR progesterone receptor[Title/Abstract] OR HR[Title/Abstract]) AND (positive[Title/Abstract] OR enriched[Title/Abstract] OR overexpressing[Title/Abstract] OR overexpressed[Title/Abstract] OR +[Title/Abstract]) AND (metastasis[Title/Abstract] OR metastatic[Title/Abstract] OR advanced[Title/Abstract] OR secondary[Title/Abstract] OR recurrent[Title/Abstract] OR inoperable[Title/Abstract] OR unresectable[Title/Abstract] OR disseminated[Title/Abstract] OR incurable[Title/Abstract] OR Stage 3[Title/Abstract] OR Stage 4[Title/Abstract] OR Stage III[Title/Abstract] OR Stage IV[Title/Abstract]) AND ((targeted therapy[Title/Abstract]) OR (CDK4/6 inhibitors[Title/Abstract] OR ulvestrant[Title/Abstract] OR ribociclib[Title/Abstract] OR abemaciclib[Title/Abstract]) OR (PI3K inhibitors[Title/Abstract] OR buparlisib[Title/Abstract] OR pictilisib[Title/Abstract] OR alpelisib[Title/Abstract] OR taselisib[Title/Abstract]) OR (AKT inhibitors[Title/Abstract] OR capivasertib[Title/Abstract] OR AZD 5363[Title/Abstract]) OR (mTOR inhibitors[Title/Abstract] OR everolimus[Title/Abstract] OR vistusertib[Title/Abstract])) AND ((endocrine therapy[Title/Abstract]) OR (selective estrogen receptor modulators[Title/Abstract] OR SERMs[Title/Abstract] OR tamoxifen[Title/Abstract] OR toremifene[Title/Abstract]) OR (selective estrogen receptor down-regulators[Title/Abstract] OR SERDs[Title/Abstract] OR ulvestrant[Title/Abstract] OR faslodex[Title/Abstract]) OR (aromatase inhibitors[Title/Abstract] OR Ais[Title/Abstract] OR letrozole[Title/Abstract] OR anatrozole[Title/Abstract] OR exemestane[Title/Abstract])) | 154 |

1. Search strategy in Embase, December 23^rd^, 2021 (read from bottom-up).

| **Set** | **Search terms** | **Result** |
| --- | --- | --- |
| #94 | #93 AND (2020:py OR 2021:py) | 514 |
| #93 | #12 AND #20 AND #25 AND #30 AND #36 AND #51 AND #91 AND #92 | 1850 |
| #92 | #74 OR #79 OR #84 OR #90 | 391695 |
| #91 | #52 OR #57 OR #63 OR #67 OR #71 | 127531 |
| #90 | #85 OR #86 OR #87 OR #88 OR #89 | 42558 |
| #89 | 'exemestane' |  |
| #88 | 'anatrozole' | 6911 |
| #87 | 'letrozole' | 6911 |
| #86 | 'ais' | 6911 |
| #85 | 'aromatase inhibitors' | 8110 |
| #84 | #80 OR #81 OR #82 OR #83 | 10432 |
| #83 | 'faslodex' | 878 |
| #82 | 'fulvestrant' | 10307 |
| #81 | 'serds' | 256 |
| #80 | 'selective estrogen receptor down-regulators' | 26 |
| #79 | #75 OR #76 OR #77 OR #78 | 73705 |
| #78 | 'toremifene' | 2355 |
| #77 | 'tamoxifen' | 71163 |
| #76 | 'serms' | 2372 |
| #75 | 'selective estrogen receptor modulators' | 2560 |
| #74 | #72 OR #73 | 302211 |
| #73 | 'endocrine therapy' | 15236 |
| #72 | 'hormonal therapy'/exp | 297922 |
| #71 | #68 OR #69 OR #70 | 37527 |
| #70 | 'vistusertib' | 479 |
| #69 | 'everolimus' | 33124 |
| #68 | 'mtor inhibitors' | 6802 |
| #67 | #64 OR #65 OR #66 | 1821 |
| #66 | 'azd 5363' | 241 |
| #65 | 'capivasertib' | 528 |
| #64 | 'akt inhibitors' | 1379 |
| #63 | #58 OR #59 OR #60 OR #61 OR #62 | 6422 |
| #62 | 'taselisib' | 358 |
| #61 | 'alpelisib' | 1318 |
| #60 | 'pictilisib' | 1213 |
| #59 | 'buparlisib' | 1956 |
| #58 | 'pi3k inhibitors' | 3149 |
| #57 | #53 OR #54 OR #55 OR #56 | 6860 |
| #56 | 'abemaciclib' | 1700 |
| #55 | 'ribociclib' | 1874 |
| #54 | 'palbociclib' | 4919 |
| #53 | 'cdk4/6 inhibitors' | 1517 |
| #52 | 'targeted therapy' | 83279 |
| #51 | #37 OR #50 | 3527812 |
| #50 | #38 OR #39 OR #40 OR #41 OR #42 OR #43 OR #44 OR #45 OR #46 OR #47 OR #48 OR #49 | 3170578 |
| #49 | 'stage iv' | 43997 |
| #48 | 'stage iii' | 67877 |
| #47 | 'stage 4' | 13365 |
| #46 | 'stage 3' | 25342 |
| #45 | 'incurable' | 20671 |
| #44 | 'disseminated' | 105308 |
| #43 | 'unresectable' | 35596 |
| #42 | 'inoperable' | 25526 |
| #41 | 'recurrent' | 599662 |
| #40 | 'secondary' | 1132626 |
| #39 | 'advanced' | 1071669 |
| #38 | 'metastatic' | 406773 |
| #37 | 'metastasis'/exp | 719793 |
| #36 | #31 OR #32 OR #33 OR #34 OR #35 | 40275911 |
| #35 | '+' | 40275911 |
| #34 | 'overexpressed' | 111164 |
| #33 | 'overexpressing' | 70496 |
| #32 | 'enriched' | 202066 |
| #31 | 'positive' | 2424471 |
| #30 | #26 OR #27 OR #28 OR #29 | 693951 |
| #29 | 'hr' | 515174 |
| #28 | 'progesterone receptor' | 48409 |
| #27 | 'estrogen receptor' | 120255 |
| #26 | 'hormone receptor' | 68341 |
| #25 | #21 OR #22 OR #23 OR #24 | 1913870 |
| #24 | '-' | 202475 |
| #23 | 'low expressed' | 930 |
| #22 | 'low expressing' | 1078 |
| #21 | 'negative' | 1721888 |
| #20 | #13 OR #19 | 154820 |
| #19 | #14 OR #15 OR #16 OR #17 OR #18 | 138723 |
| #18 | 'human epidermal growth factor receptor 2' | 19505 |
| #17 | 'erbb2' | 15900 |
| #16 | 'neu' | 66169 |
| #15 | 'her2' | 71053 |
| #14 | 'her-2' | 14358 |
| #13 | epidermal growth factor receptor 2'/exp | 65341 |
| #12 | #1 OR #11 | 744688 |
| #11 | #9 AND #10 | 741657 |
| #10 | #4 OR #5 OR #6 OR #7 OR #8 | 6401780 |
| #9 | #2 OR #3 | 922199 |
| #8 | 'tumor' | 3307640 |
| #7 | 'neoplasm' | 945592 |
| #6 | 'malignancy' | 285120 |
| #5 | 'carcinoma' | 1298613 |
| #4 | 'cancer' | 4681436 |
| #3 | 'mammary' | 103077 |
| #2 | 'breast' | 883671 |
| #1 | 'breast cancer'/exp | 518954 |

1. Search strategy in the Cochrane Library, December 23^rd^, 2021.

| **Set** | **Search terms** | **Result** |
| --- | --- | --- |
| #1 | MeSH descriptor: [Breast Neoplasms] explode all trees | 14397 |
| #2 | (breast):ti,ab,kw (Word variations have been searched) | 52697 |
| #3 | (mammary):ti,ab,kw (Word variations have been searched) | 1434 |
| #4 | (cancer):ti,ab,kw (Word variations have been searched) | 178827 |
| #5 | (carcinoma):ti,ab,kw (Word variations have been searched) | 44124 |
| #6 | (malignancy):ti,ab,kw (Word variations have been searched) | 29018 |
| #7 | (neoplasm):ti,ab,kw (Word variations have been searched) | 87192 |
| #8 | (tumor):ti,ab,kw (Word variations have been searched) | 79933 |
| #9 | #2 OR #3 | 53429 |
| #10 | #4 OR #5 OR #6 OR #7 OR #8 | 237124 |
| #11 | #9 AND #10 | 41594 |
| #12 | #1 OR #11 | 41594 |
| #13 | MeSH descriptor: [Receptor, ErbB-2] 7 tree(s) exploded | 974 |
| #14 | (HER-2):ti,ab,kw (Word variations have been searched) | 816 |
| #15 | (HER2):ti,ab,kw (Word variations have been searched) | 6319 |
| #16 | (neu):ti,ab,kw (Word variations have been searched) | 921 |
| #17 | (ERBB2):ti,ab,kw (Word variations have been searched) | 353 |
| #18 | (human epidermal growth factor receptor 2):ti,ab,kw (Word variations have been searched) | 3602 |
| #19 | #13 OR #14 OR #15 OR #16 OR #17 OR #18 | 8909 |
| #20 | (negative):ti,ab,kw (Word variations have been searched) | 90989 |
| #21 | (low expressing):ti,ab,kw (Word variations have been searched) | 10986 |
| #22 | (low expressed):ti,ab,kw (Word variations have been searched) | 10986 |
| #23 | #20 OR #21 OR #22 | 100543 |
| #24 | (hormone receptor):ti,ab,kw (Word variations have been searched) | 8798 |
| #25 | (estrogen receptor):ti,ab,kw (Word variations have been searched) | 5071 |
| #26 | (progesterone receptor):ti,ab,kw (Word variations have been searched) | 1986 |
| #27 | (HR):ti,ab,kw (Word variations have been searched) | 49568 |
| #28 | #24 OR #25 OR #26 OR #27 | 59159 |
| #29 | (positive):ti,ab,kw (Word variations have been searched) | 178688 |
| #30 | (enriched):ti,ab,kw (Word variations have been searched) | 8669 |
| #31 | (overexpressing):ti,ab,kw (Word variations have been searched) | 2111 |
| #32 | (overexpressed):ti,ab,kw (Word variations have been searched) | 2160 |
| #33 | #29 OR #30 OR #31 OR #32 | 187548 |
| #34 | MeSH descriptor: [Neoplasm Metastasis] explode all trees | 5451 |
| #35 | (metastasis):ti,ab,kw (Word variations have been searched) | 21985 |
| #36 | (metastatic):ti,ab,kw (Word variations have been searched) | 29691 |
| #37 | (advanced):ti,ab,kw (Word variations have been searched) | 73773 |
| #38 | (secondary):ti,ab,kw (Word variations have been searched) | 301005 |
| #39 | (recurrent):ti,ab,kw (Word variations have been searched) | 78313 |
| #40 | (inoperable):ti,ab,kw (Word variations have been searched) | 2743 |
| #41 | (unresectable):ti,ab,kw (Word variations have been searched) | 6132 |
| #42 | (disseminated):ti,ab,kw (Word variations have been searched) | 10580 |
| #43 | (incurable):ti,ab,kw (Word variations have been searched) | 2733 |
| #44 | (Stage 3):ti,ab,kw (Word variations have been searched) | 58544 |
| #45 | (Stage 4):ti,ab,kw (Word variations have been searched) | 46944 |
| #46 | (Stage III):ti,ab,kw (Word variations have been searched) | 20853 |
| #47 | (Stage IV):ti,ab,kw (Word variations have been searched) | 15431 |
| #48 | #34 OR #35 OR #36 OR #37 OR #38 OR #39 OR #40 OR #41 OR #42 OR #43 OR #44 OR #45 OR #46 OR #47 | 468173 |
| #49 | (targeted therapy):ti,ab,kw (Word variations have been searched) | 51514 |
| #50 | (CDK4/6 inhibitors):ti,ab,kw (Word variations have been searched) | 339 |
| #51 | (palbociclib):ti,ab,kw (Word variations have been searched) | 480 |
| #52 | (ribociclib):ti,ab,kw (Word variations have been searched) | 238 |
| #53 | (abemaciclib):ti,ab,kw (Word variations have been searched) | 218 |
| #54 | #50 OR #51 OR #52 OR #53 | 959 |
| #55 | (PI3K inhibitors):ti,ab,kw (Word variations have been searched) | 591 |
| #56 | (buparlisib):ti,ab,kw (Word variations have been searched) | 66 |
| #57 | (pictilisib):ti,ab,kw (Word variations have been searched) | 20 |
| #58 | (alpelisib):ti,ab,kw (Word variations have been searched) | 97 |
| #59 | (taselisib):ti,ab,kw (Word variations have been searched) | 45 |
| #60 | #55 OR #56 OR #57 OR #58 OR #59 | 677 |
| #61 | (AKT inhibitors):ti,ab,kw (Word variations have been searched) | 448 |
| #62 | (capivasertib):ti,ab,kw (Word variations have been searched) | 34 |
| #63 | (AZD 5363):ti,ab,kw (Word variations have been searched) | 0 |
| #64 | #61 OR #62 OR #63 | 462 |
| #65 | (mTOR inhibitors):ti,ab,kw (Word variations have been searched) | 844 |
| #66 | (everolimus):ti,ab,kw (Word variations have been searched) | 4250 |
| #67 | (vistusertib):ti,ab,kw (Word variations have been searched) | 17 |
| #68 | #65 OR #66 OR #67 | 4657 |
| #69 | #49 OR #54 OR #60 OR #64 OR #68 | 56103 |
| #70 | (endocrine therapy):ti,ab,kw (Word variations have been searched) | 6259 |
| #71 | (selective estrogen receptor modulators):ti,ab,kw (Word variations have been searched) | 885 |
| #72 | (SERMs):ti,ab,kw (Word variations have been searched) | 302 |
| #73 | (tamoxifen):ti,ab,kw (Word variations have been searched) | 5059 |
| #74 | (toremifene):ti,ab,kw (Word variations have been searched) | 208 |
| #75 | (selective estrogen receptor down-regulators):ti,ab,kw (Word variations have been searched) | 18 |
| #76 | (SERDs):ti,ab,kw (Word variations have been searched) | 38 |
| #77 | (fulvestrant):ti,ab,kw (Word variations have been searched) | 863 |
| #78 | (faslodex):ti,ab,kw (Word variations have been searched) | 129 |
| #79 | (aromatase inhibitors):ti,ab,kw (Word variations have been searched) | 2383 |
| #80 | (AIs):ti,ab,kw (Word variations have been searched) | 4728 |
| #81 | (letrozole):ti,ab,kw (Word variations have been searched) | 2291 |
| #82 | (anatrozole):ti,ab,kw (Word variations have been searched) | 2 |
| #83 | (exemestane):ti,ab,kw (Word variations have been searched) | 953 |
| #84 | #70 OR #71 OR #72 OR #73 OR #74 OR #75 OR #76 OR #77 OR #78 OR #79 OR #80 OR #81 OR #82 OR #83 | 17454 |
| #85 | #12 AND #19 AND #23 AND #28 AND #33 AND #48 AND #69 AND #84 | 221 |

**Supplementary Figure 1** Overall comparisons between CKD4/6i or PI3K/AKT/mTORi and endocrine therapy.

A.


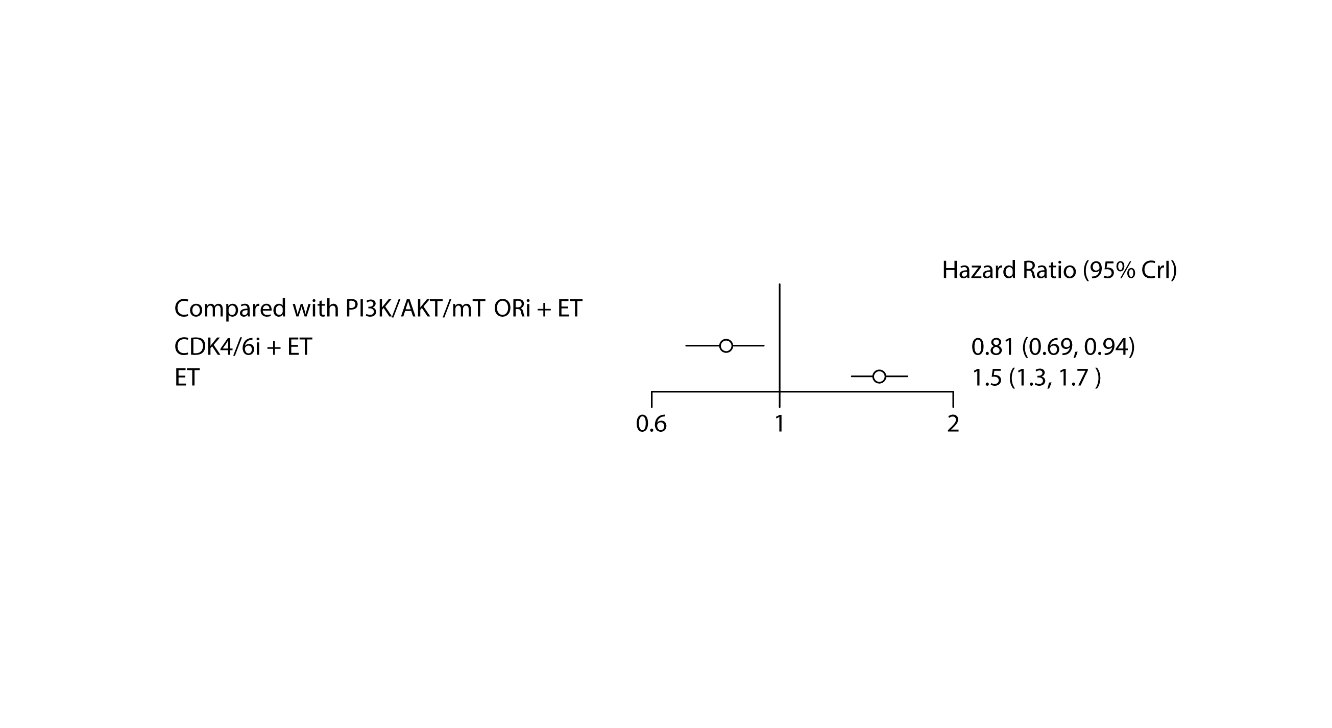


B.


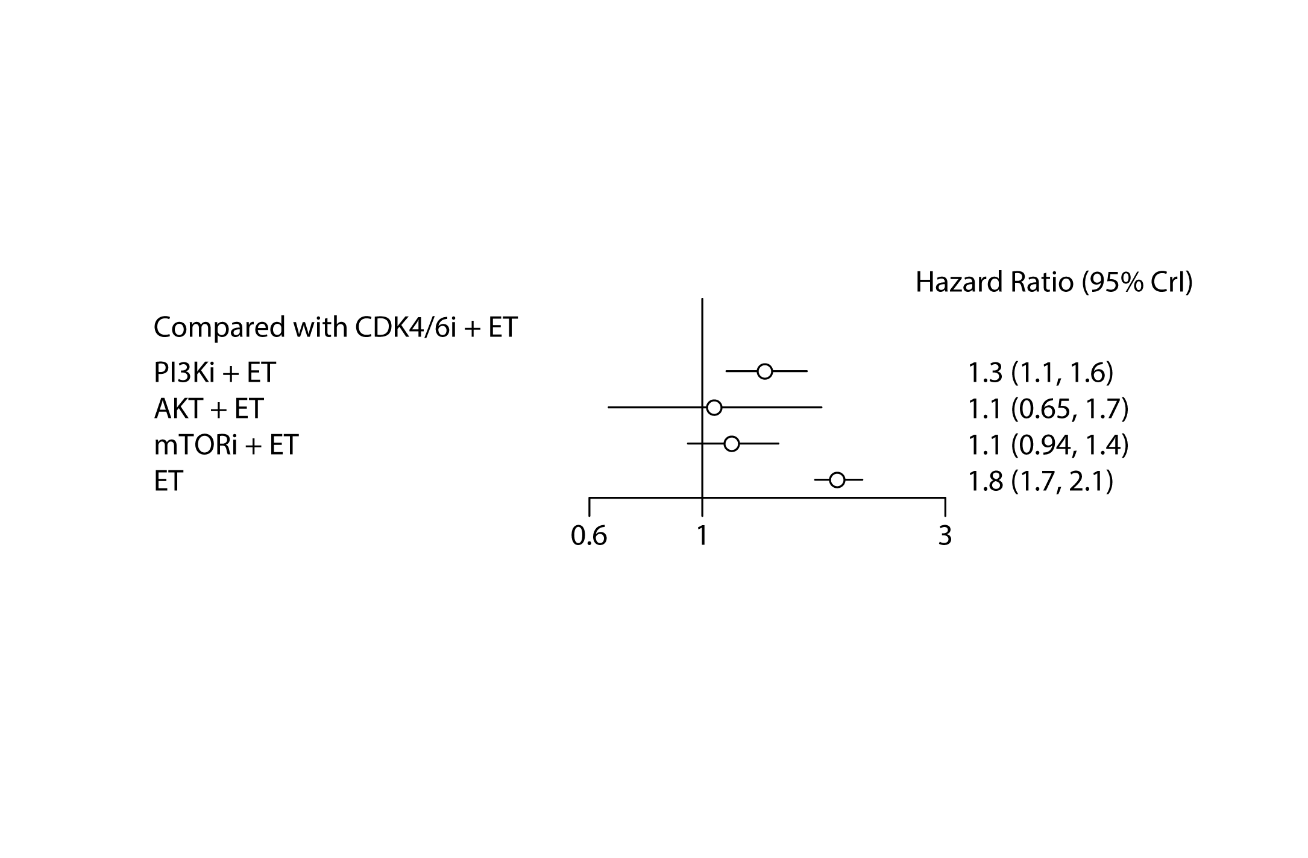


C.


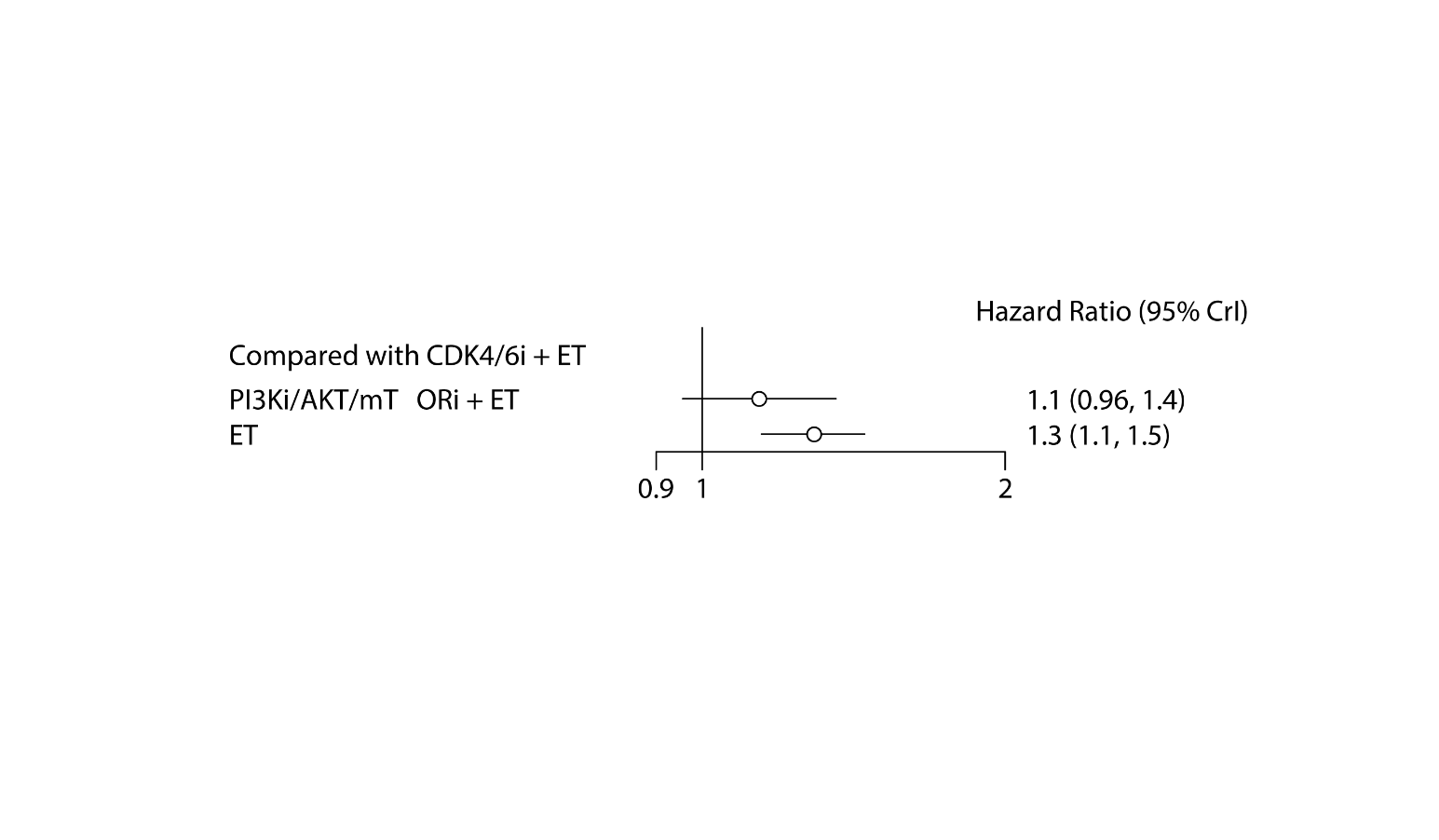


D.


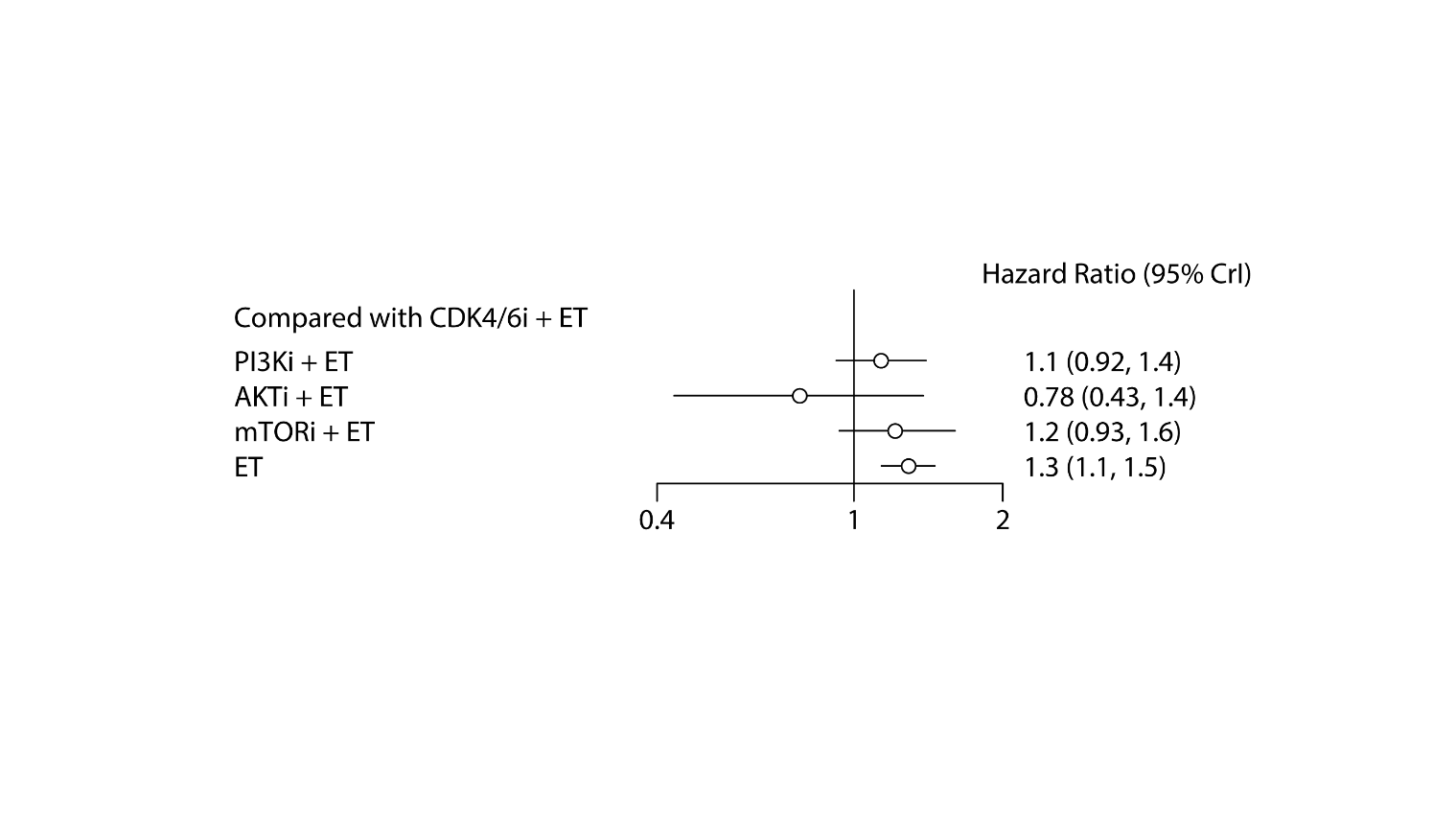


1. PFS comparisons between CDK4/6 inhibitors and PI3K/AKT/mTOR inhibitors. (I^2^=6%)
2. PFS comparisons among PI3K inhibitors, AKT inhibitors, mTOR inhibitors and CDK4/6 inhibitors. (I^2^=8%)
3. OS comparisons between CDK4/6 inhibitors and PI3K/AKT/mTOR inhibitors. (I^2^=6%)
4. OS comparisons among PI3K inhibitors, AKT inhibitors, mTOR inhibitors and CDK4/6 inhibitors. (I^2^=0%)

**Supplementary Figure 2** Comparisons between CDK4/6 inhibitors and PI3K/AKT/mTOR inhibitors when stratified by treatment lines

A.


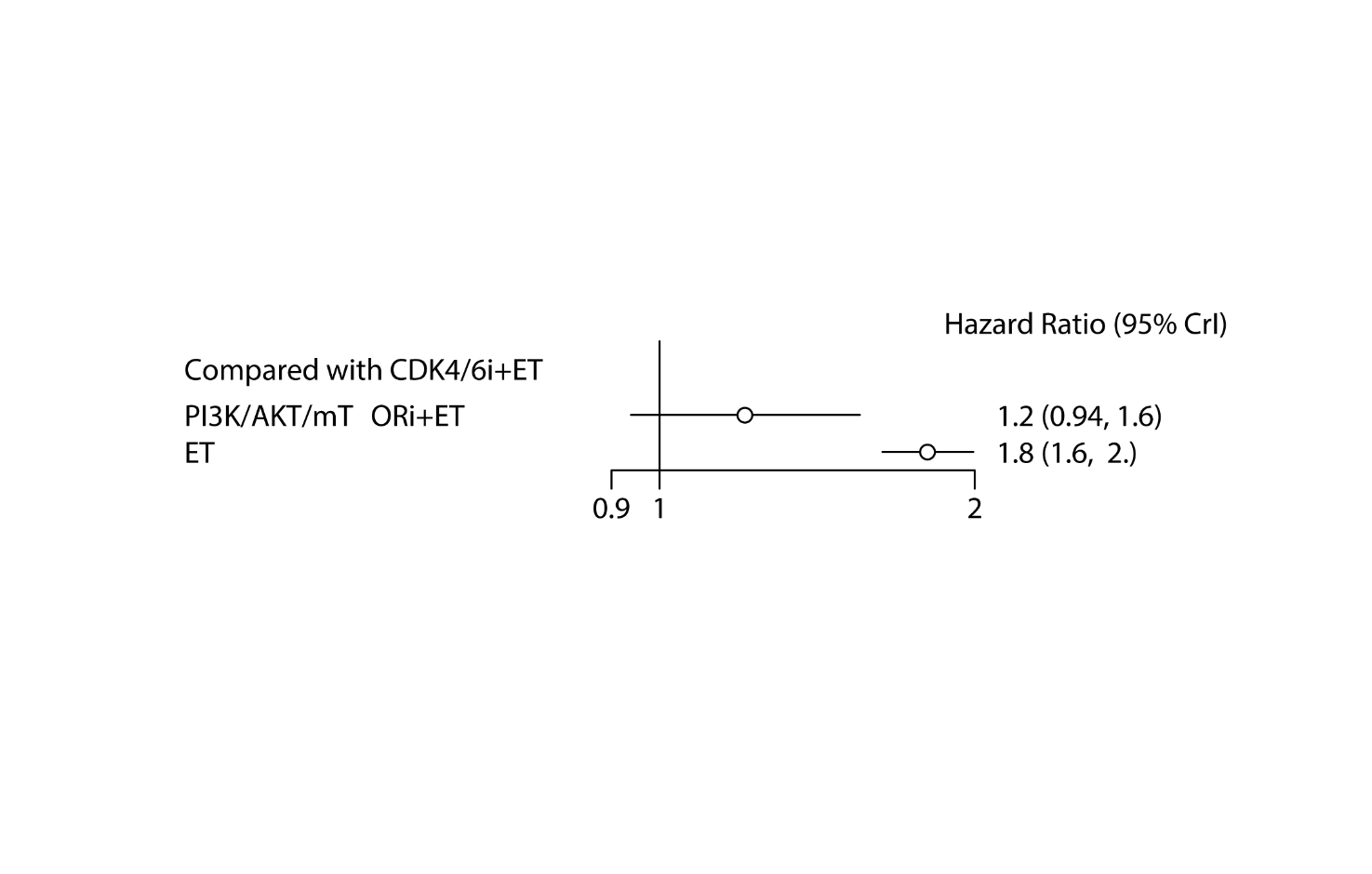


B.


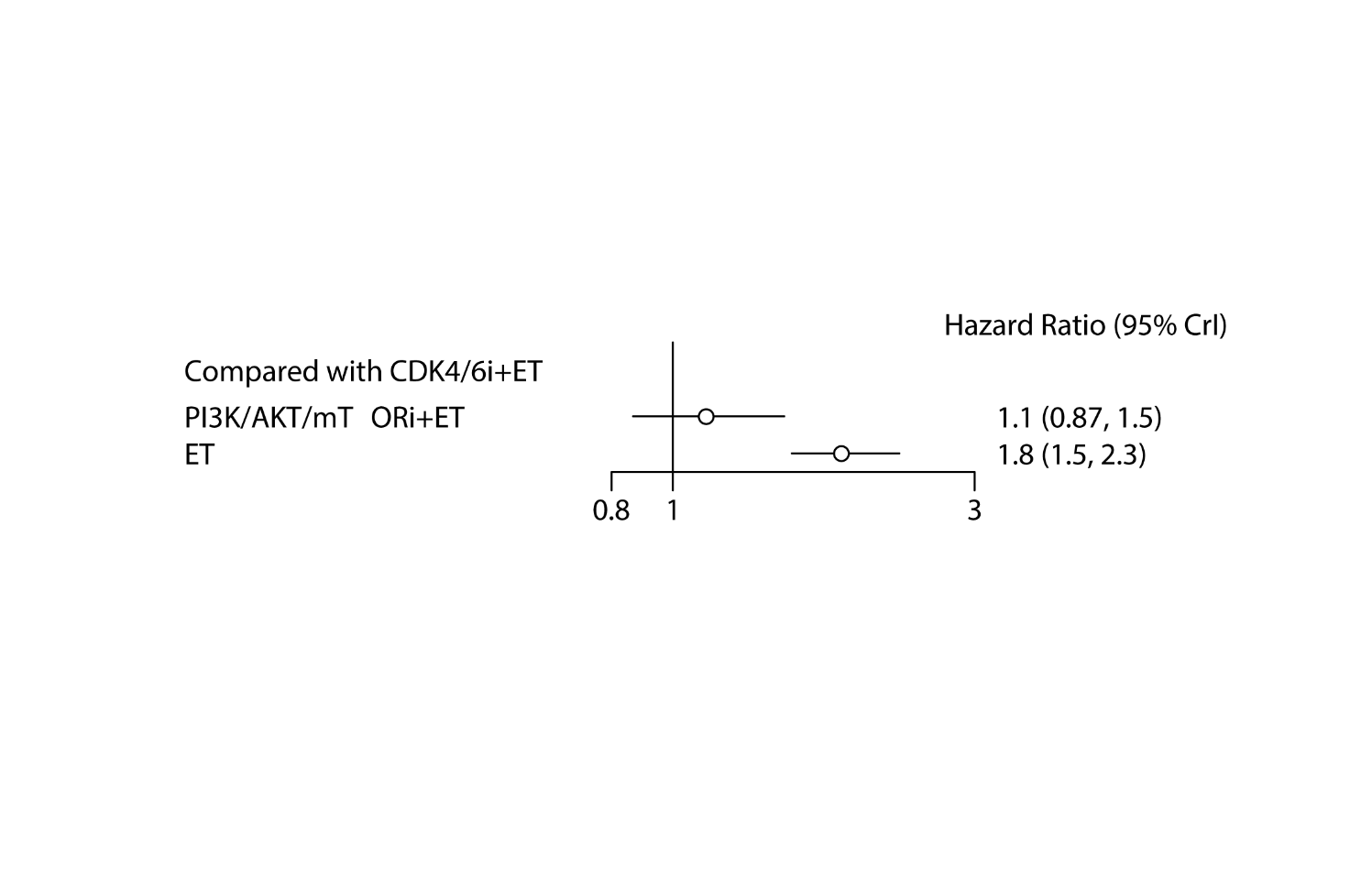


C.


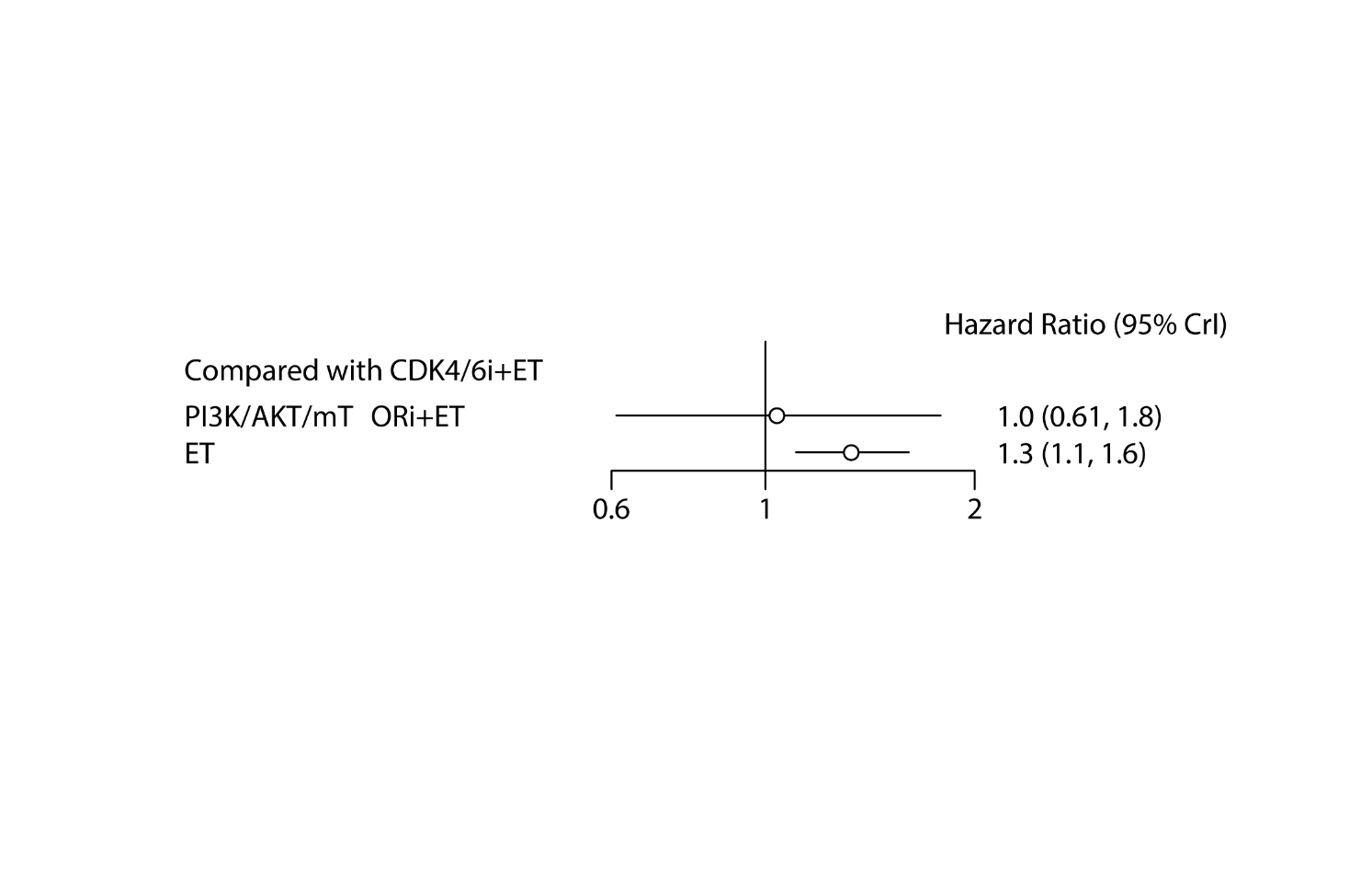


D.


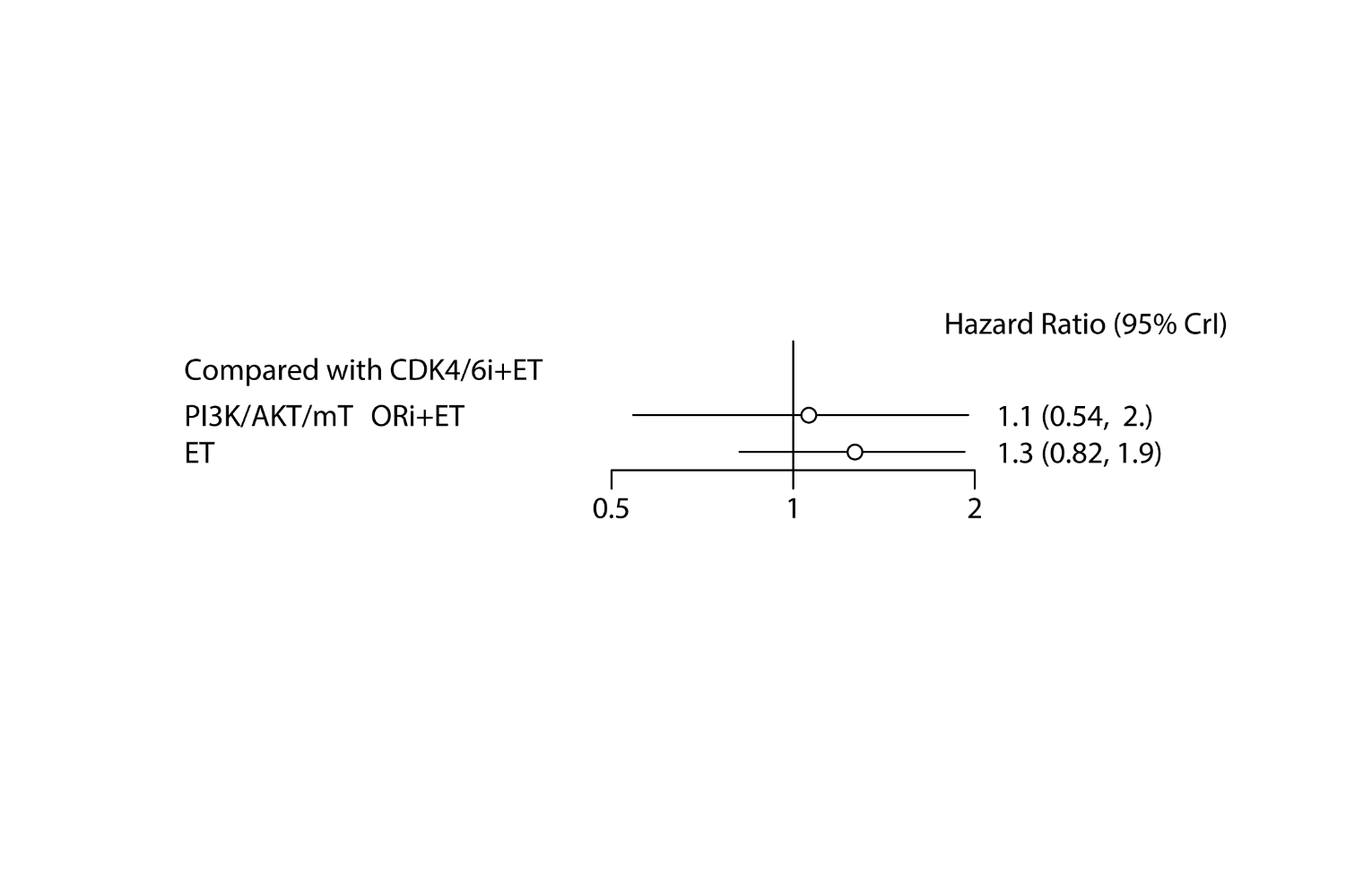


1. PFS comparisons for first line therapy. (I^2^=0%)
2. PFS comparisons for second line therapy. (I^2^=0%)
3. OS comparisons for first line therapy. (I^2^=0%)
4. OS comparisons for second line therapy. (I^2^=32%)

**Supplementary Figure 3** PFS comparisons between CDK4/6 inhibitors and PI3K/AKT/mTOR inhibitors in visceral **(A)** and non-visceral metastasis subgroups **(B)** (I^2^=0% and 14%).

A.


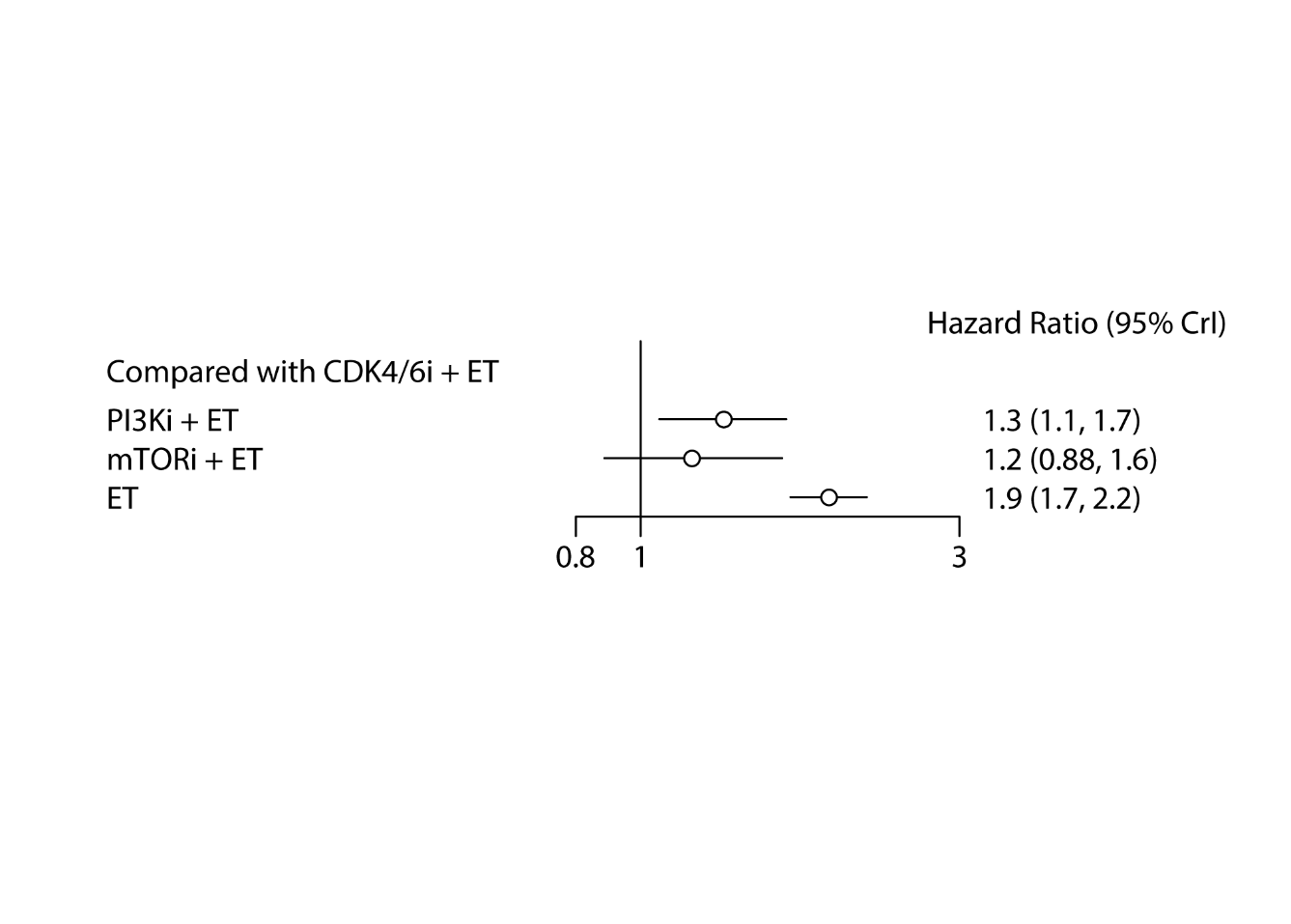


B.


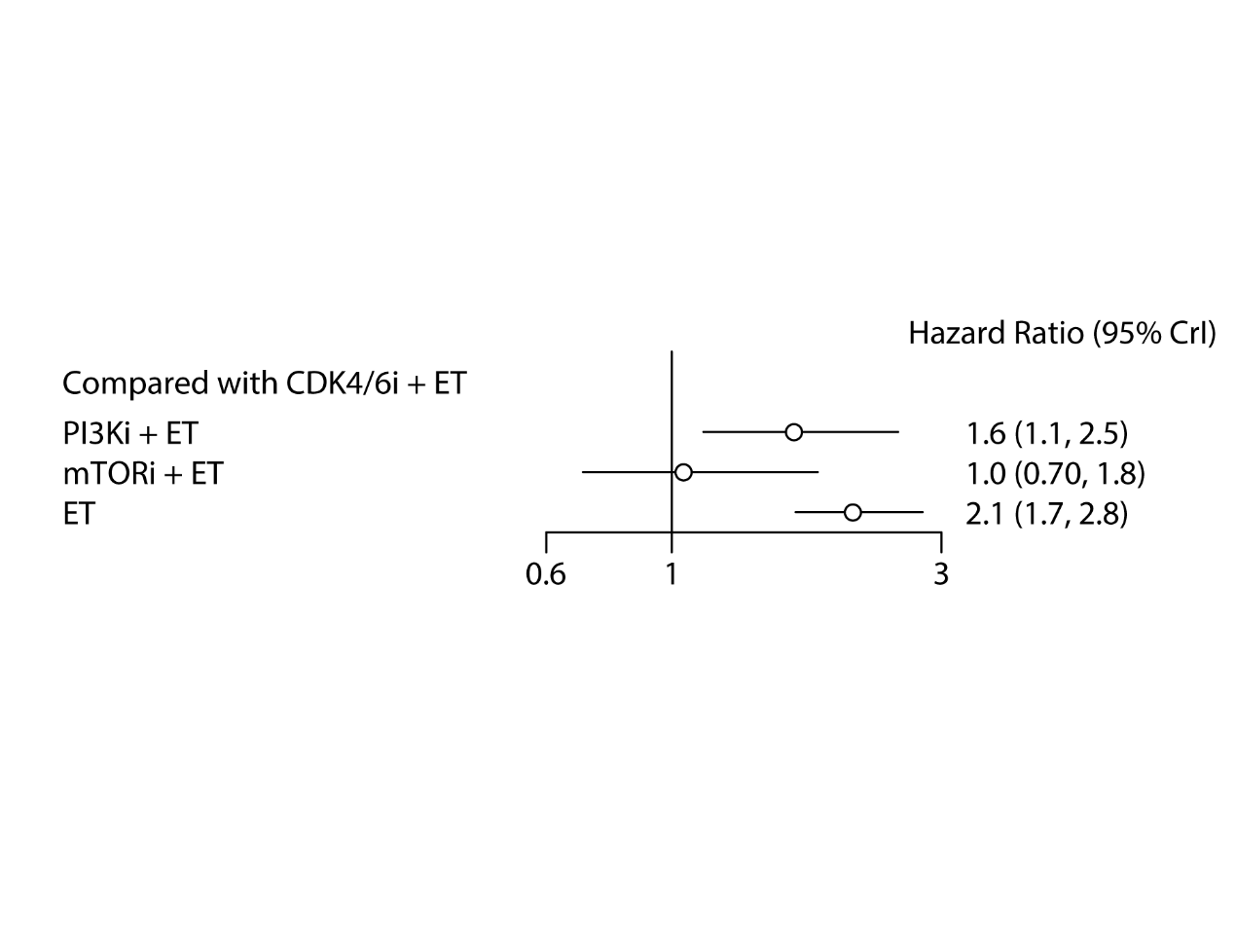


**Supplementary** **Figure 4** PFS comparisons between CDK4/6 inhibitors and PI3K/AKT/mTOR inhibitors in bone-only metastasis subgroup. (I^2^=11%)


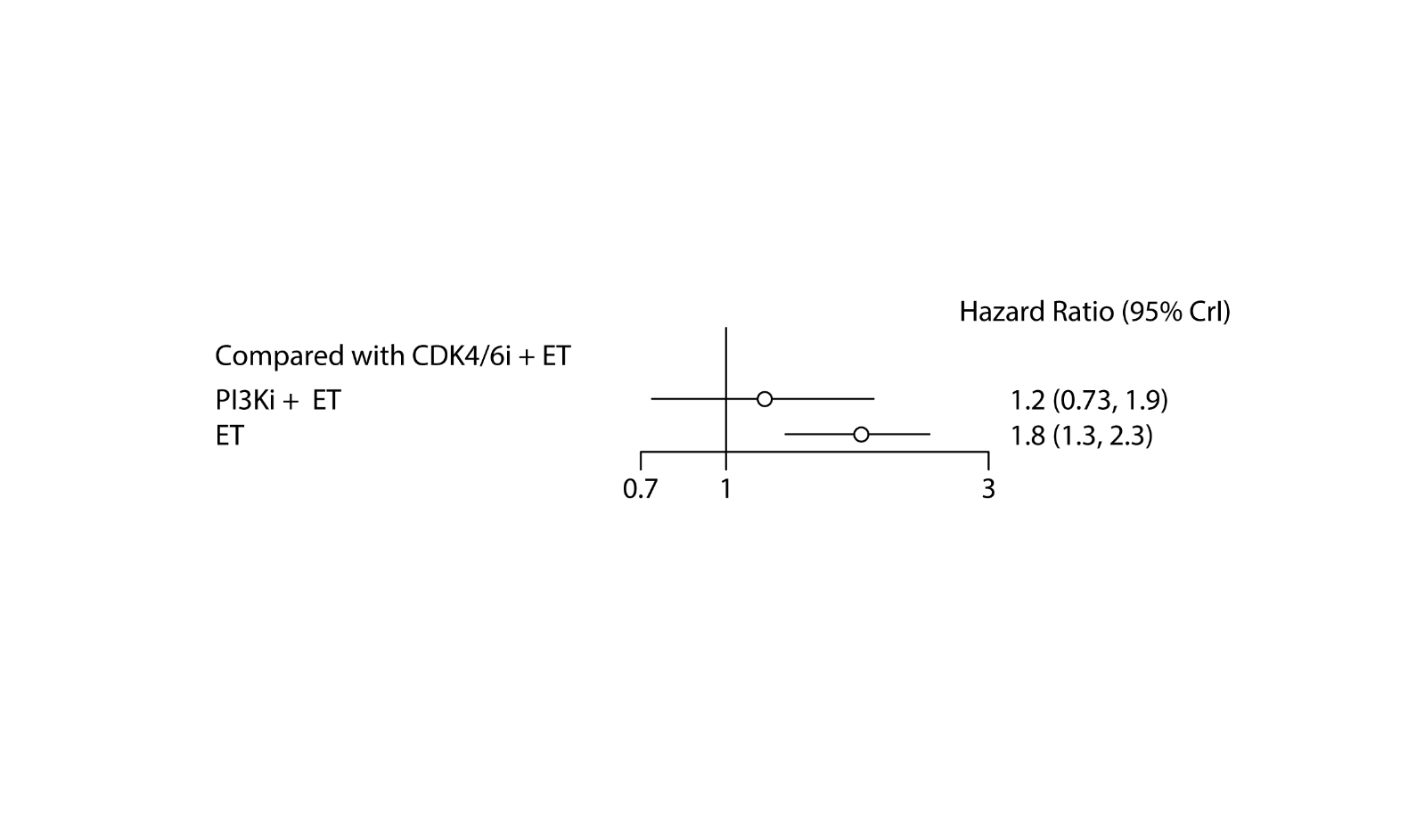


**Supplementary** **Figure 5** PFS comparisons between CDK4/6 inhibitors and PI3K/AKT/mTOR inhibitors in liver metastasis subgroup. (I^2^=0%)


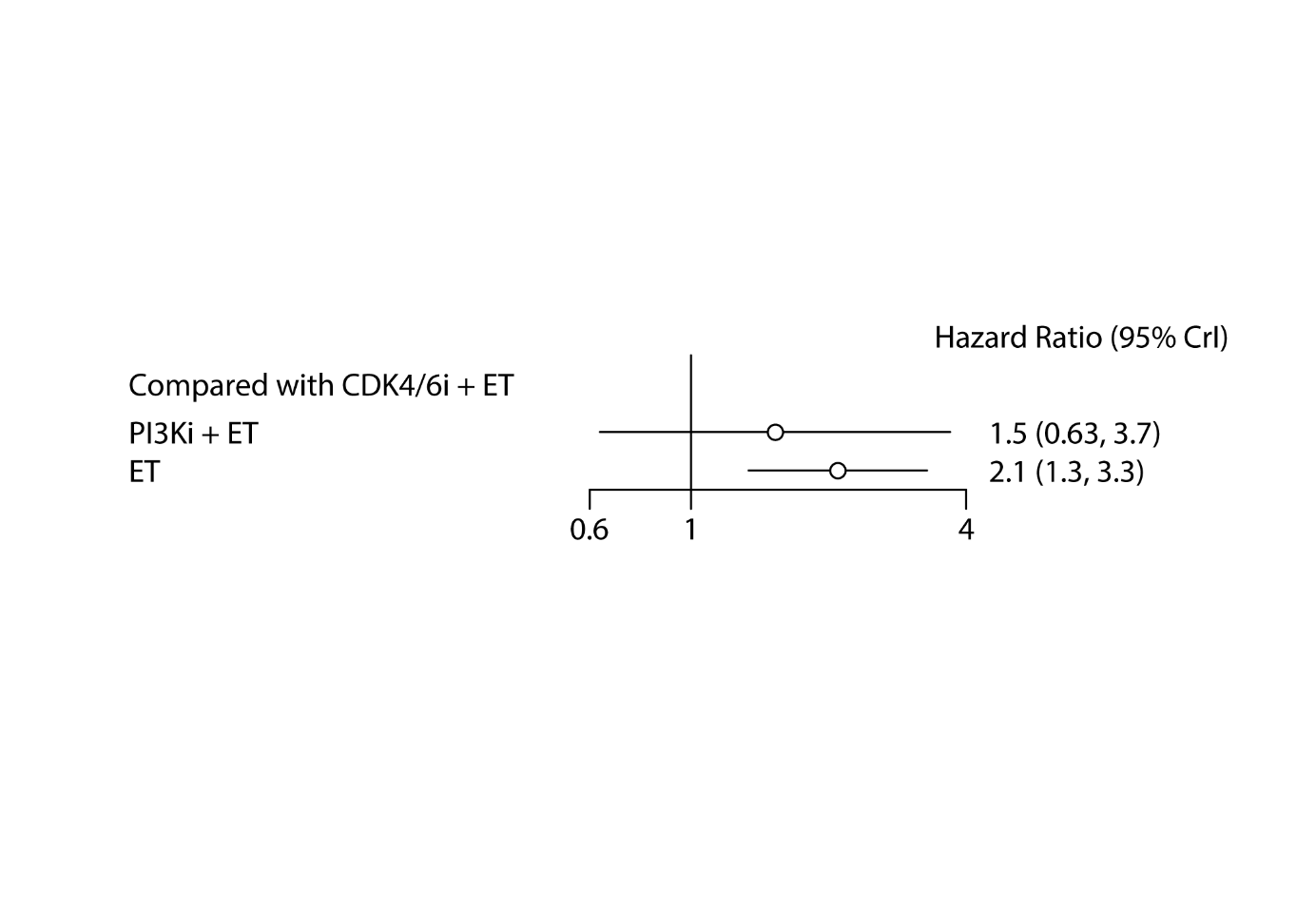


**Supplementary** **Figure 6** Time to first subsequent chemotherapy comparisons between CDK4/6 inhibitors and PI3K inhibitors. (I^2^=17%)


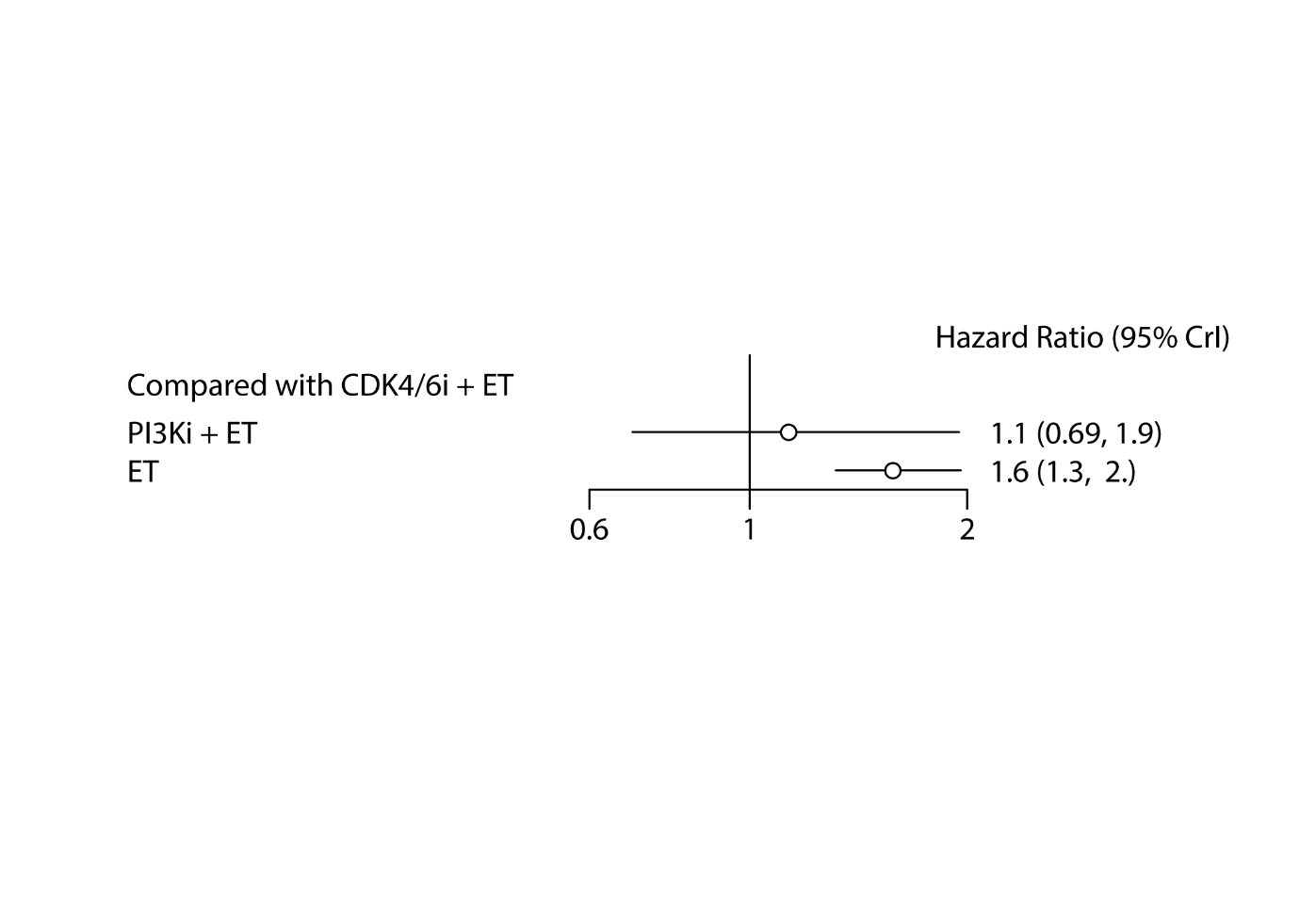


**Supplementary** **Figure 7** ≥3 grade neutropenia comparisons within CDK4/6 inhibitors. (I^2^=7%)


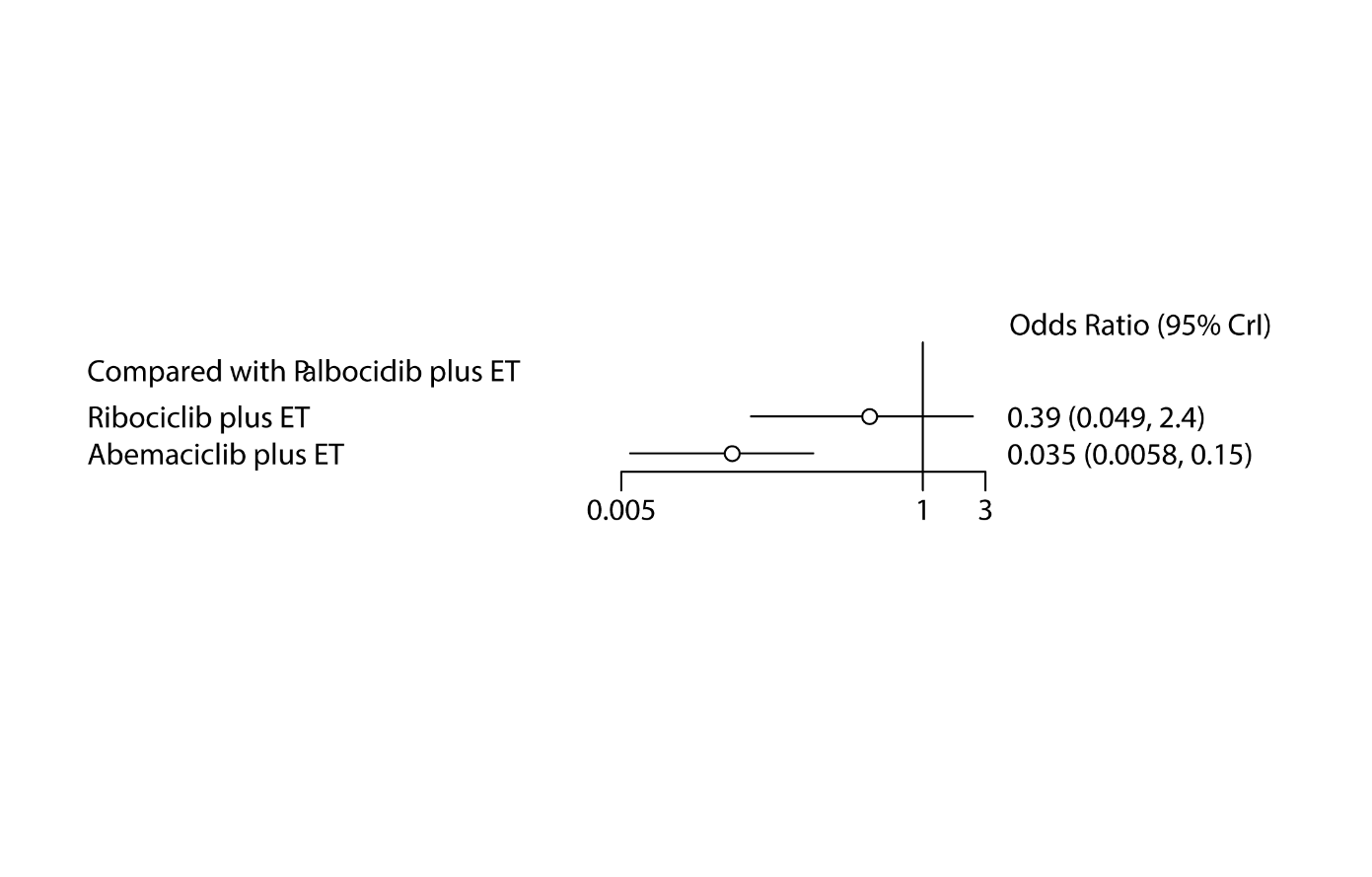


**Supplementary** **Figure 8** All grade stomatitis comparisons within PI3K/AKT/mTOR inhibitors. (I^2^=4%)


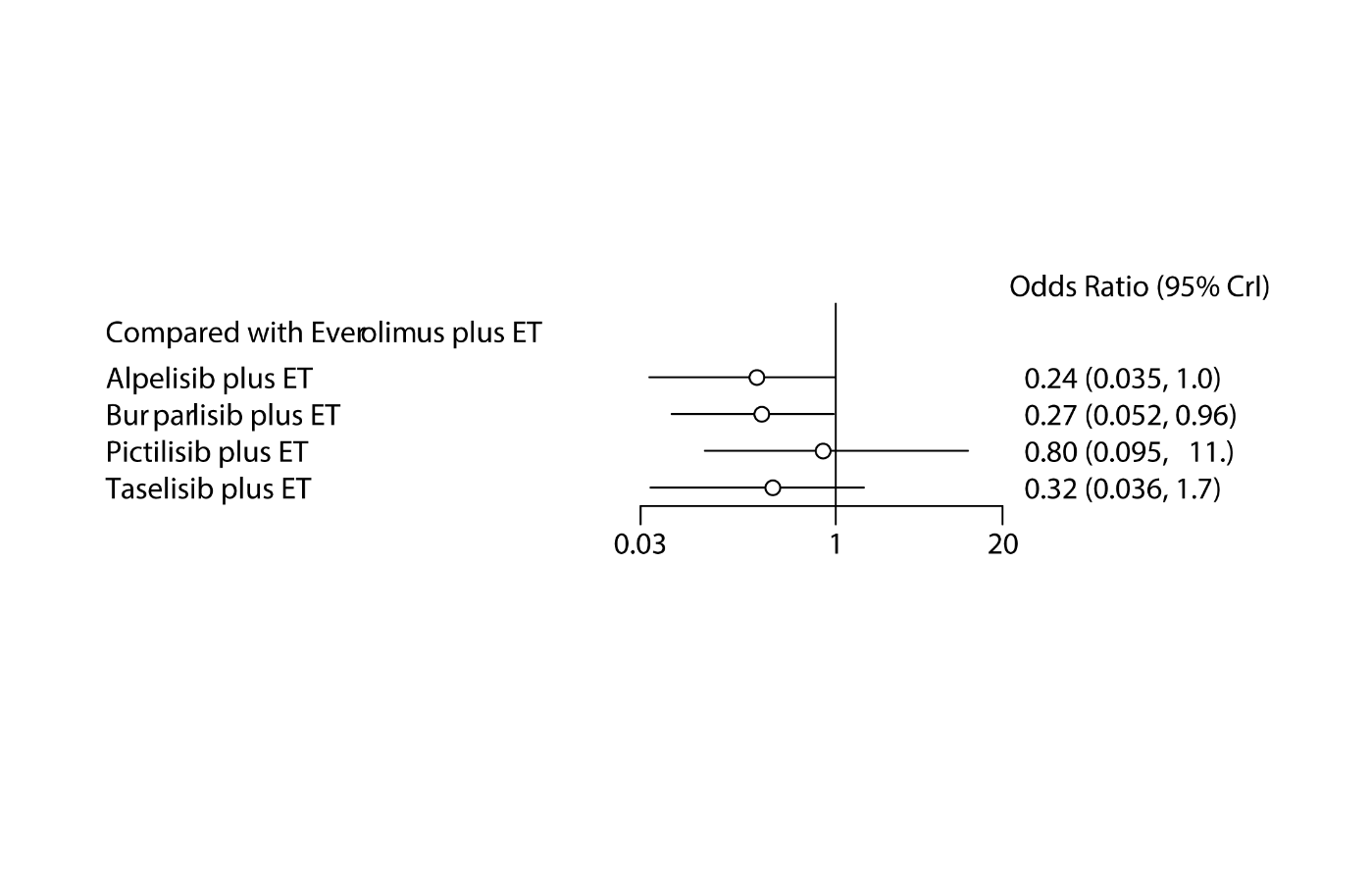


**Supplementary** **Figure 9** All grade nausea comparisons within PI3K/AKT/mTOR inhibitors. (I^2^=8%)


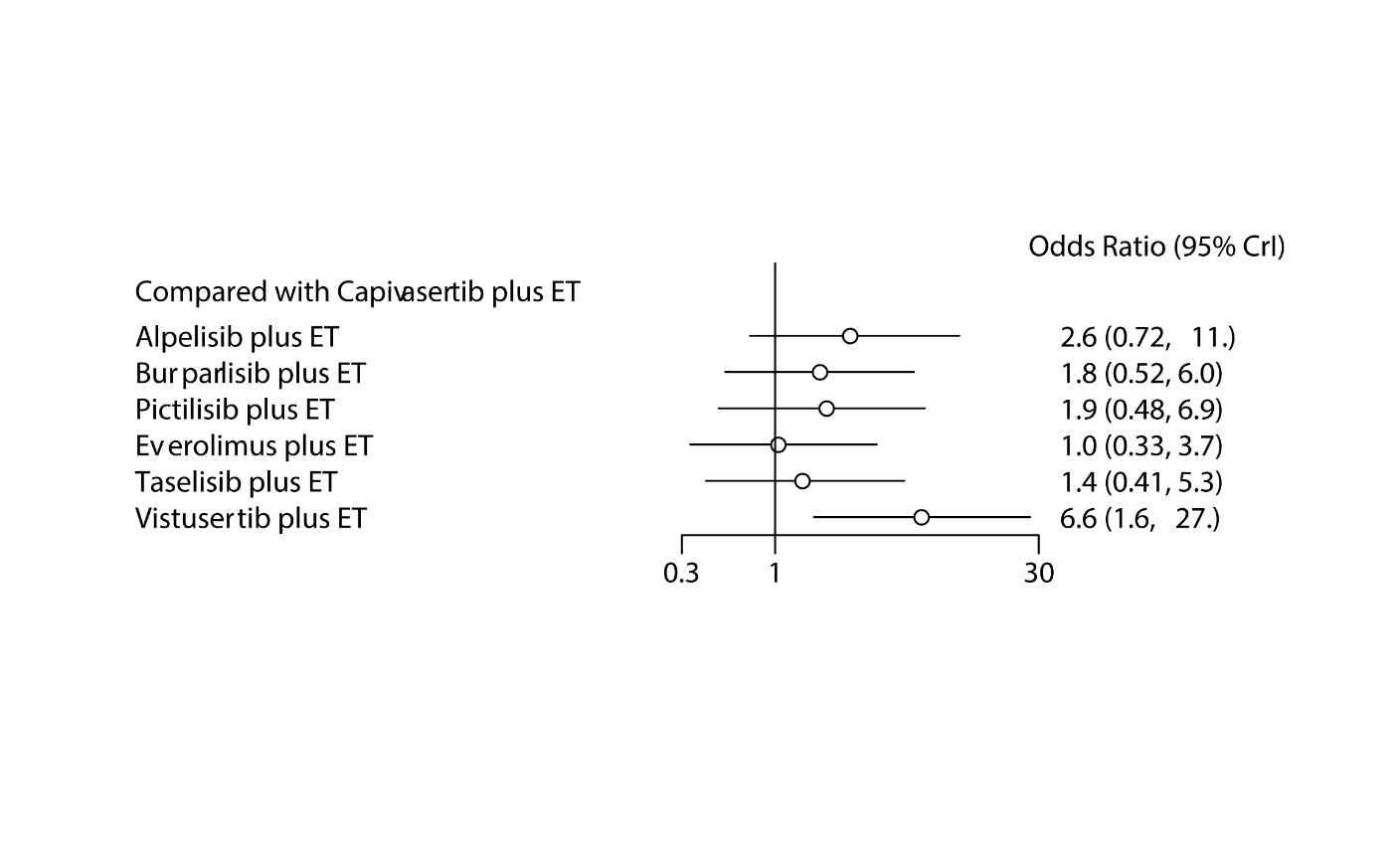


**Supplementary** **Figure 10** All grade anorexia comparisons within PI3K/AKT/mTOR inhibitors. (I^2^=0%)


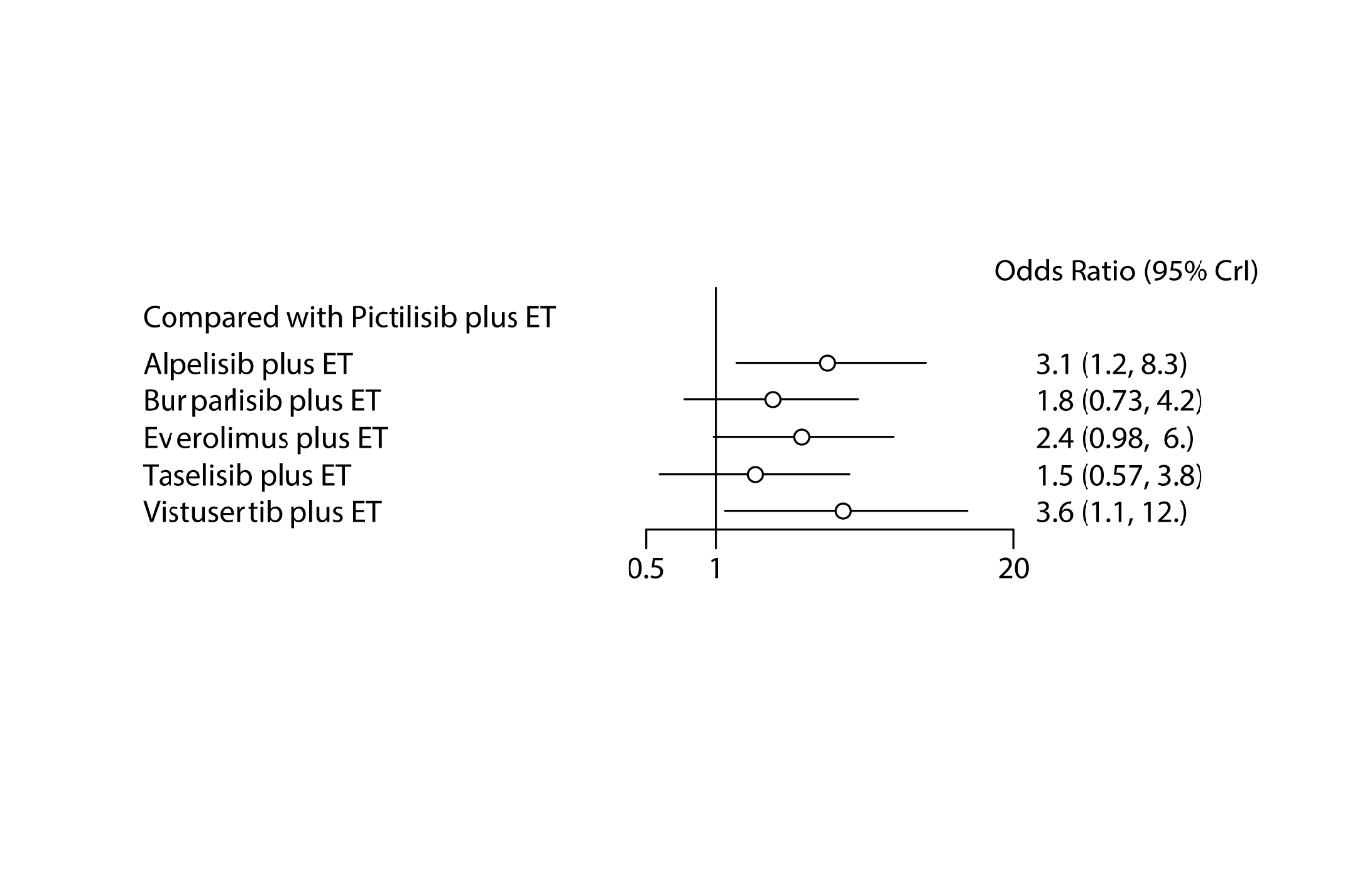


**Supplementary** **Figure 11** All grade elevated ALT concentration comparisons among all treatments. (I^2^=0.4%)


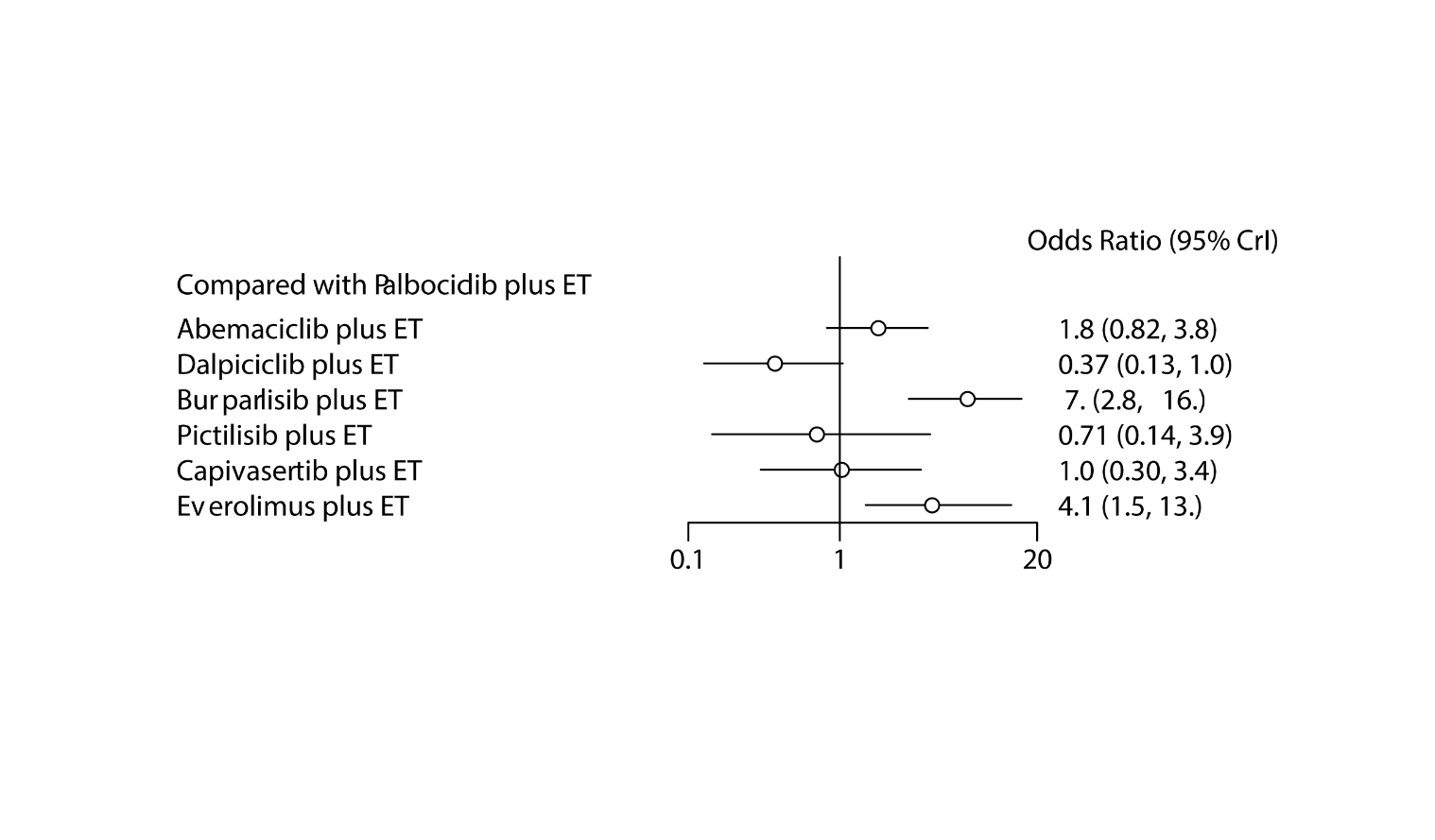


**Supplementary** **Figure 12** All grade elevated AST concentration comparisons among all treatments. (I^2^=0%)


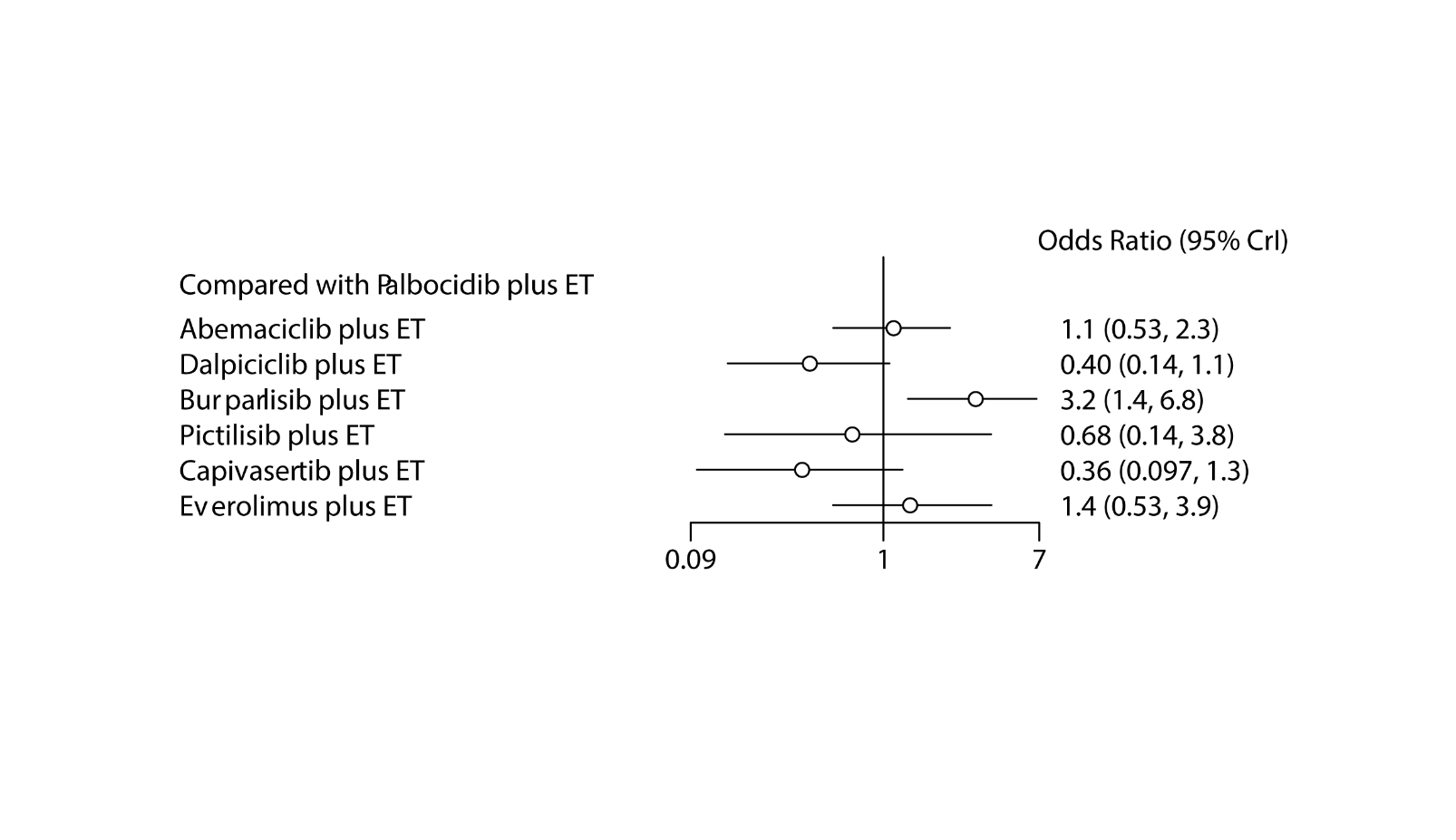


**Supplementary** **Figure 13** All grade diarrhea comparisons among all treatments. (I^2^=5%)


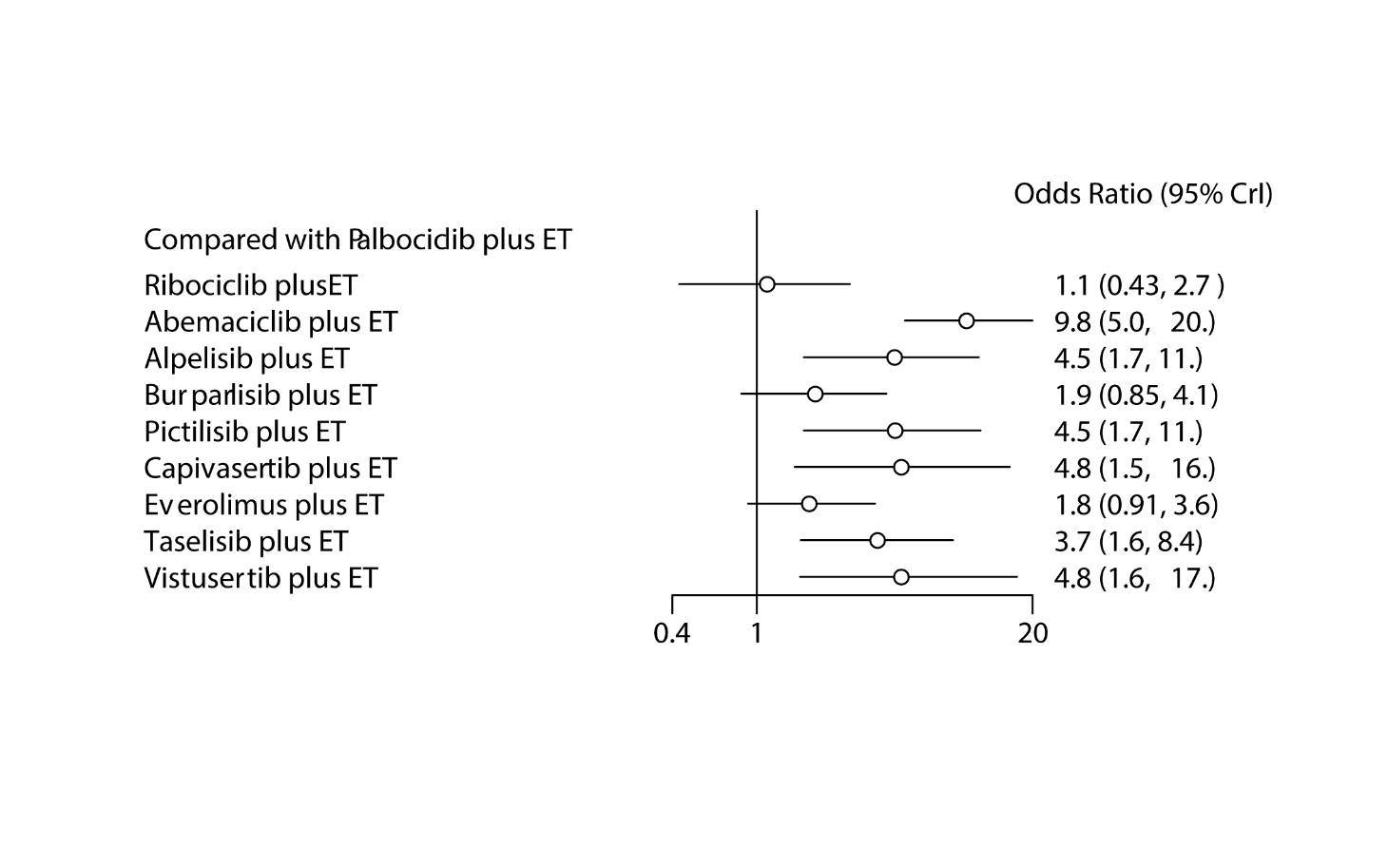


**Supplementary Figure 14** All grade hyperglycemia comparisons among all treatments. (I^2^=4%)


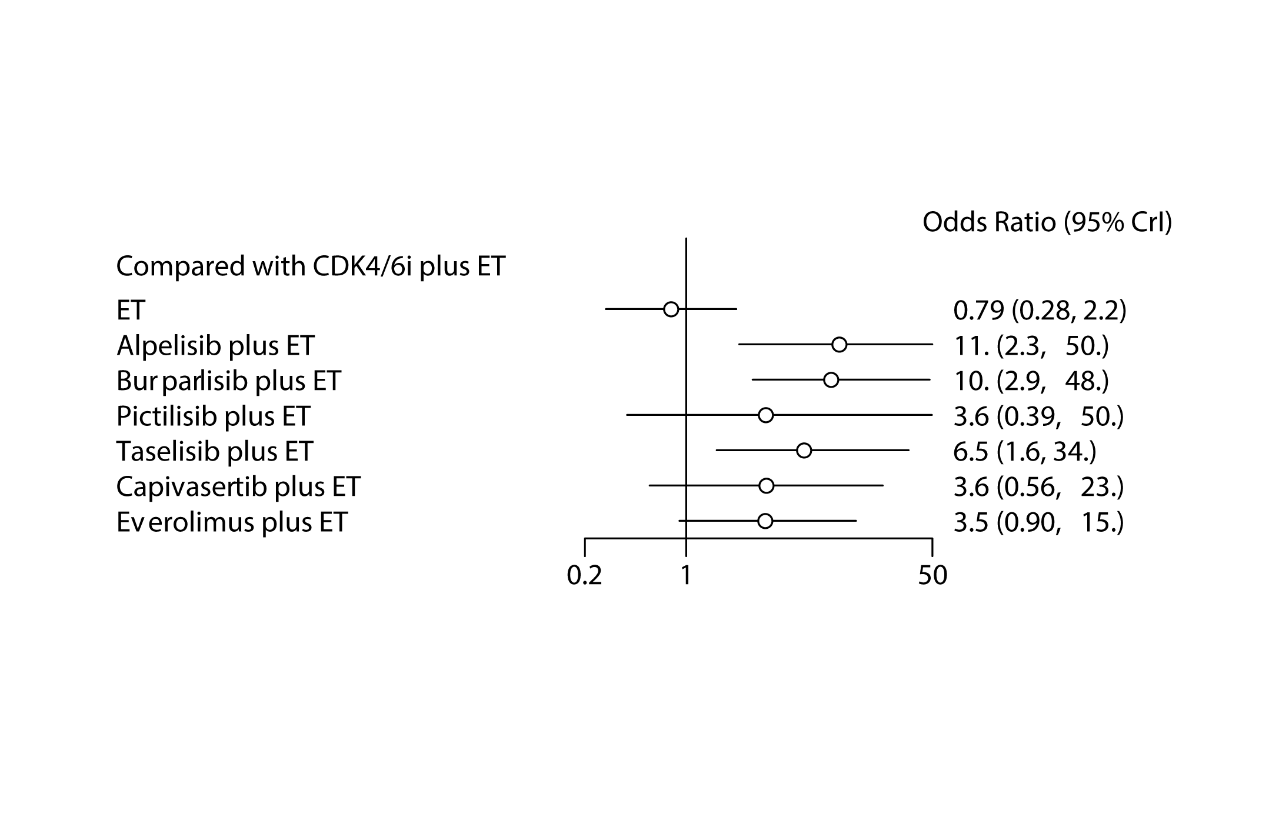

Supplement: Supplementary Table 1 — Full search strategy in (A) PubMed, (B) Embase and (C) the Cochrane Library. [file DataSheet_1.docx]
